# Clofarabine, cytarabine, and mitoxantrone in refractory/relapsed acute myeloid leukemia: High response rates and effective bridge to allogeneic hematopoietic stem cell transplantation

**DOI:** 10.1002/cam4.2865

**Published:** 2020-03-18

**Authors:** Harinder Gill, Rita Yim, Herbert H. Pang, Paul Lee, Thomas S. Y. Chan, Yu‐Yan Hwang, Garret M. K. Leung, Ho‐Wan Ip, Rock Y. Y. Leung, Sze‐Fai Yip, Bonnie Kho, Harold K. K. Lee, Vivien Mak, Chi‐Chung Chan, June S. M. Lau, Chi‐Kuen Lau, Shek‐Yin Lin, Raymond S. M. Wong, Wa Li, Edmond S. K. Ma, Jun Li, Gianni Panagiotou, Joycelyn P. Y. Sim, Albert K. W. Lie, Yok‐Lam Kwong

**Affiliations:** ^1^ Department of Medicine The University of Hong Kong Hong Kong SAR China; ^2^ School of Public Health The University of Hong Kong Hong Kong SAR China; ^3^ Department of Pathology Queen Mary Hospital Hong Kong SAR China; ^4^ Department of Medicine Tuen Mun Hospital Hong Kong SAR China; ^5^ Department of Medicine Pamela Youde Nethersole Eastern Hospital Hong Kong SAR China; ^6^ Department of Medicine Princess Margaret Hospital Hong Kong SAR China; ^7^ Department of Medicine Queen Elizabeth Hospital Hong Kong SAR China; ^8^ Department of Medicine Tseung Kwan O Hospital Hong Kong SAR China; ^9^ Department of Medicine United Christian Hospital Hong Kong SAR China; ^10^ Department of Medicine and Therapeutics Prince of Wales Hospital Hong Kong SAR China; ^11^ Department of Clinical Oncology Prince of Wales Hospital Hong Kong SAR China; ^12^ Department of Pathology Hong Kong Sanatorium and Hospital Hong Kong SAR China; ^13^ Department of Infectious Diseases and Public Health The City University of Hong Kong Hong Kong SAR China; ^14^ School of Biological Sciences The University of Hong Kong Hong Kong SAR China; ^15^ Department of Microbiology Li Ka Shing Faculty of Medicine The University of Hong Kong Hong Kong SAR China; ^16^ Department of Systems Biology and Bioinformatics Leibniz Institute for Natural Product Research and Infection Biology Hans Knöll Institute (HKI) Jena Germany

**Keywords:** acute myeloid leukemia, adult, clofarabine, cytarabine, mitoxantrone, refractory, relapsed

## Abstract

Clofarabine is active in refractory/relapsed acute myeloid leukemia (AML). In this phase 2 study, we treated 18‐ to 65‐year‐old AML patients refractory to first‐line 3 + 7 daunorubicin/cytarabine induction or relapsing after 3 + 7 induction and high‐dose cytarabine consolidation, with clofarabine (30 mg/m^2^/d, Days 1‐5), cytarabine (750 mg/m^2^/d, Days 1‐5), and mitoxantrone (12 mg/m^2^/d, Days 3‐5) (CLAM). Patients achieving remission received up to two consolidation cycles of 50% CLAM, with eligible cases bridged to allogeneic hematopoietic stem cell transplantation (allo‐HSCT). The mutational profile of a 69‐gene panel was evaluated. Twenty‐six men and 26 women at a median age of 46 (22‐65) years were treated. The overall response rate after the first cycle of CLAM was 90.4% (complete remission, CR: 69.2%; CR with incomplete hematologic recovery, CRi: 21.2%). Twenty‐two CR/CRi patients underwent allo‐HSCT. The 2‐year overall survival (OS), relapse‐free survival (RFS), and event‐free survival (EFS) were 65.8%, 45.7%, and 40.2%, respectively. Multivariate analyses showed that superior OS was associated with CR after CLAM (*P* = .005) and allo‐HSCT (*P* = .005), and superior RFS and EFS were associated with allo‐HSCT (*P* < .001). Remarkably, CR after CLAM and allo‐HSCT resulted in 2‐year OS of 84.3% and 90%, respectively. Karyotypic aberrations and genetic mutations did not influence responses or survivals. Grade 3/4 neutropenia/thrombocytopenia and grade 3 febrile neutropenia occurred in all cases. Other nonhematologic toxicities were mild and uncommon. There was no treatment‐related mortality and the performance of allo‐HSCT was not compromised. Clofarabine, cytarabine, and mitoxantrone was highly effective and safe in refractory/relapsed AML. This study was registered at ClinicalTrials.gov (NCT02686593).

## Graphical abstract

Fifty‐two patients with relapsed/refractory acute myeloid leukemia (AML) were treated with clofarabine, cytarabine, and mitoxantrone. The overall response rate (ORR) was 90.4% (complete remission, CR: 69.2%; CR with incomplete hematological recovery, CRi: 21.2%), with 22 CR/CRi patients undergoing allogeneic hematopoietic stem cell transplantation (allo‐HSCT), while in CR; resulting in a 2‐year OS of 84.3% in CR patients and 90% in patients receiving allo‐HSCT. Clofarabine, cytarabine, and mitoxantrone resulted in high ORR and was safe in refractory/relapsed AML.

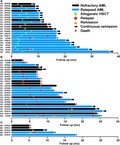

## 1 INTRODUCTION

Standard daunorubicin/cytarabine induction for newly‐diagnosed acute myeloid leukemia (AML) results in a first complete remission (CR1) rate of 60‐85%.^1^ For induction failures, salvage with other intensive regimens leads to poor overall response rates (ORR) of <40%.^2^ For patients achieving CR1, long‐term disease‐free survival is only 30%‐40%, implying that relapses occur in the majority of cases.^3^ The outcome of relapsed AML is poor with conventional chemotherapeutic approaches, which resulted in ORRs of merely 16%‐21%.^4^, ^5^ Significant improvements were not achieved with newer drugs in relapsed AML (ORRs, vosaroxin: 30%^4^; elacytarabine: 23%^5^). Recently, drugs targeting specific genetic mutations have shown promises.^6^ However, such approaches are only applicable to a small fraction of patients and durable disease‐free survivals are uncommonly achieved. Hence, current strategies for refractory/relapsed AML aim at achieving a response without excessive toxicities, so that eligible patients can undergo allogeneic hematopoietic stem cell transplantation (HSCT), which may be curative.

Clofarabine, a structural hybrid of fludarabine and cladribine, is a second‐generation nucleoside analog with potent antileukemic effects^7^; owing to its ability to inhibit ribonucleotide reductase and DNA polymerase, induce apoptosis, and enhance intracellular accumulation of cytarabine.^8^ Early studies showed that clofarabine possessed significant activities in AML, as either salvage therapy or initial treatment for patients unsuitable for intensive chemotherapy.^9^, ^10^, ^11^, ^12^, ^13^ These results ushered in a plethora of studies combining clofarabine with cytarabine, daunorubicin, idarubicin, mitoxantrone, etoposide, and amsacrine in refractory/relapsed and newly diagnosed AML.^14^, ^15^, ^16^, ^17^, ^18^, ^19^, ^20^, ^21^, ^22^, ^23^, ^24^, ^25^, ^26^, ^27^, ^28^, ^29^, ^30^, ^31^, ^32^, ^33^, ^34^, ^35^, ^36^ In refractory/relapsed AML, clofarabine combinations have given modest ORRs of 36%‐61.5%.^9^, ^17^, ^18^, ^20^, ^27^, ^36^

In a previous study of refractory/relapsed AML, we combined clofarabine (40 mg/m^2^/d × 5) with high‐dose cytarabine (1‐2 g/m^2^/d × 5), observing an ORR of 43%.^16^ However, infective complications, particularly invasive fungal diseases, were significant at such high doses of clofarabine and cytarabine.^37^ Given the observed safety and potential synergism between clofarabine and anthracyclines/anthracenedione, we evaluated the effectiveness and safety of combining lower doses of clofarabine and cytarabine with mitoxantrone (CLAM). The objectives were to preserve efficacy and decrease infective complications, so that subsequent allogeneic HSCT might not be compromised.

## 2 PATIENTS AND METHODS

### 2.1 Patients

This was an investigator‐initiated phase 2 study recruiting adult (18‐65 year old) patients with AML refractory to first‐line “3 + 7” induction (3 days of daunorubicin at 90 mg/m^2^/d for 18‐ to 60‐year‐old patients and 60 mg/m^2^/d for 61‐ to 65‐year‐old patients; 7 days of cytarabine at 100 mg/m^2^/d) (defined as failure to achieve complete remission, CR; or CR with incomplete hematologic recovery, CRi; after one cycle of 3 + 7 induction); or in first relapse (R1) during CR1 after “3 + 7” induction and high‐dose cytarabine consolidation (4 g/m^2^/d × 2, four monthly cycles). Eligibility criteria included marrow blasts >5% at recruitment and an Eastern Cooperative Oncology Group performance score of 0‐1. The main exclusion criteria included acute promyelocytic leukemia, uncontrolled infection, significant cardiac impairment and arrhythmia, renal and liver dysfunction, and central nervous system leukemia. Pathology and karyotypes at diagnosis and relapse were centrally reviewed (HWI, RYYL) according to the World Health Organization 2016 classification.^38^, ^39^, ^40^ The study was approved by the Institute Review Board and registered at ClinicalTrials.gov (NCT02686593). All patients gave written informed consent and the study was performed according to the Declaration of Helsinki.

### 2.2 Sample size calculation

In our center, the ORR of refractory/relapsed AML to conventional salvage therapies (FLAG, fludarabine, cytarabine, G‐CSF; MDAC/MTZ, medium‐dose cytarabine, mitoxantrone; ICE: idarubicin, cytarabine, etoposide) was about 25%. To give a 20% improvement, a sample size of ≥50 patients was required to give at least 80% power (2‐sided α level = 0.05).

### 2.3 Treatment

Clofarabine, cytarabine, and mitoxantrone comprised clofarabine (Sanofi) (30 mg/m^2^/d, intravenous infusion, IV, over 1 hour, Days 1‐5), cytarabine (750 mg/m^2^/d, IV over 2 hours, starting 4 hours after clofarabine, Days 1‐5), and mitoxantrone (EBEWE Pharma GmbH) (12 mg/m^2^/d, IV over 1 hour, Days 3‐5) (details of the CLAM protocol can be found in File S1). Bone marrow aspiration and trephine biopsy were performed on Day 28. Patients with response (marrow <5% blasts) received a maximum of 2 cycles of CLAM consolidation, each at 50% dose reduction, given 6‐8 weeks apart. Responding patients with an HLA‐matched sibling or volunteer‐unrelated donor were offered allogeneic HSCT.

### 2.4 Evaluations and supportive care

Transthoracic echocardiogram for left ventricular ejection fraction was performed before each cycle of CLAM. Granulocyte colony‐stimulating factor was started on Day 6 and administered until the neutrophil count was ≥1 × 10^9^/L. Quantitative polymerase chain reaction (Q‐PCR) for plasma cytomegalovirus was monitored weekly. Cytomegalovirus reactivation was treated preemptively with ganciclovir (IV, 250 mg/d) until Q‐PCR was negative. All patients received antifungal prophylaxis with posaconazole (oral, 200 mg thrice daily), voriconazole (oral, 200 mg/d) or micafungin (IV, 100 mg/d), depending on liver function; and anti‐pneumocystis prophylaxis with oral co‐trimoxazole or inhalational pentamidine.

### 2.5 Adverse events

The National Cancer Institute Common Terminology Criteria for Adverse Events version 4.0^41^ were adopted. During chemotherapy, all grade 3 nonhematologic toxicities mandated temporary treatment cessation until recovery to ≤grade 2. In between chemotherapy, all grade 3 hematologic or nonhematologic toxicities mandated delay of the next chemotherapy until recovery to ≤grade 2. Toxicities lasting >8 weeks or any grade 4 toxicity, mandated study withdrawal. For patients undergoing allogeneic HSCT, complications including acute and chronic graft‐vs‐host disease (GVHD) were evaluated as previously described.^42^

### 2.6 Next‐generation sequencing

Targeted next‐generation sequencing (NGS) was performed on DNA samples from diagnostic bone marrow aspirates in all cases. A custom xGen Lockdown Panel targeting 69 myeloid‐relevant genes (File S2) was designed based on GRCh37/hg19 (Integrated DNA Technologies). All exons of the 69 genes were sequenced, with a total of 2885 probes covering 273.03 kb. The enriched libraries were sequenced pair‐ended with the Illumina MiSeq System (Illumina). FASTQ files containing at least 1 million raw reads with coverage of 500× were generated for bioinformatic analyses as described^43^ (File S2).

### 2.7 End‐points and definitions

The primary end‐point was the response after the first cycle of CLAM as assessed by bone marrow examination on Day 28. Complete remission and CRi were defined according to standard criteria.^40^ Nonremission (NR) was defined as bone marrow blasts ≥5% following the first cycle of CLAM. Secondary end‐points were overall survival (OS), relapse‐free survival (RFS), event‐free survival (EFS), and AEs. Overall survival was defined as the time from the start of CLAM to death from any cause (event) or the latest follow‐up (censor). RFS was defined as the time from CR/CRi after the first cycle of CLAM to relapse (bone marrow blasts ≥5%, circulating blasts or development of extramedullary disease) (event), death from any cause (event) or latest follow‐up (censor). Event‐free survival was defined as the time from the start of CLAM to treatment failure (event), relapse from CR/CRi (event), death (event) or latest follow‐up (censor). The duration of response (DOR), not itself an end‐point, was calculated from the time of first response (CR/CRi) to time of loss of response, death from all causes, HSCT or latest follow‐up, whichever the earliest.

### 2.8 Statistical analyses

Analyses of categorical variables were performed with the Fisher's exact and *χ* ^2^ tests, and continuous variables with the Mann‐Whitney *U* or Kruskal‐Wallis test. Survivals were estimated with the Kaplan‐Meier method. Differences in survivals were compared with the logrank test and Cox proportional hazard model. Additional censoring for survivals was not performed at allogeneic HSCT. Prognostic impacts on response were evaluated for the following parameters: gender, age (18‐45 years vs 46‐65 years), prior status (“3 + 7”‐refractory vs R1), and gene mutations (occurring in ≥5% of patients) (wild type vs mutated). These parameters, together with response after CLAM (CR vs CRi vs NR) and allogeneic HSCT (for responding patients only) (performed vs not performed) were evaluated for impact on survivals. Parameters with *P* ≤ .1 on univariate analysis were further evaluated by multivariate analysis. Statistical analyses were performed using SPSS version 23.0 (Chicago, IL, USA). *P* values (2‐tailed) of <.05 were considered significant.

## 3 RESULTS

### 3.1 Patients

Between February 1, 2016, and March 31, 2018, 26 men and 26 women at a median age of 46 (22‐65) years were recruited from eight regional hospitals (Table 1). At initial diagnosis, karyotypes were normal in 25 cases and abnormal in 27 cases, of which 10 were considered to confer an inferior prognosis^40^ (File S3). These included inv(3)(q21.3q26.2)/t(3;3)(q21.2;q23.3) (N = 6), t(v;11q23.3)/del(11)(q23) (N = 2) and complex karyotypes (N = 2). Status before CLAM was “3 + 7”‐refractory (N = 21) and R1 (N = 31). For R1 patients, relapse occurred at a median of 12 (2‐53) months from CR1. Data were censored on January 31, 2019.

**Table 1:** Clinicopathologic characteristics of 52 patients with relapsed or refractory acute myeloid leukemia treated with CLAM

Characteristics	Value
Gender	
Male	26
Female	26
Age	
18‐45 y	24
46‐65 y	28
Median age, y (range)	46 (22‐65)
Characteristics at diagnosis	
Median leucocyte count, ×10^9^/L (range)	11.4 (0.59‐377)
Median hemoglobin, g/dL (range)	8.1 (3.9‐12.3)
Median platelet count, ×10^9^/L (range)	70 (8‐433)
Median marrow blast percentage (range)	68.5 (20‐96)
Karyotype	
Normal	25
Abnormal	27
t(8;21)(q22;q22.1); *RUNX1‐RUNX1T1*	7
inv(16)(p13.1q22)/t(16;16)(p13.1;q22); *CBFB‐MYH11*	2
t(9;11)(p21.2;q23.3); *MLLT3‐KMT2A*	1
inv(3)(q21.3q26.2)/t(3;3)(q21.2;q23.3); *GATA2‐MECOM(EVI1)*	6
t(v;11q23.3)/ del(11)(q23); *KMT2A* rearranged	2
Complex^a^	2
Others	7
Characteristics at the start of CLAM	
Nonremission after first induction	21
First relapse	31
Median time to first relapse, mo (range)	12 (2‐53)
>12 mo from CR1	12
≤12 mo from CR1	19
Median leucocyte count, ×10^9^/L (range)	2.92 (0.26‐99.8)
Median hemoglobin, g/dL (range)	9.95 (7.7‐14.5)
Median platelet count, ×10^9^/L (range)	74.5 (5‐407)
Median marrow blast percentage (range)	60 (6‐94)
Median duration of follow‐up, mo (range)	15 (4‐36)

Abbreviations: CLAM, clofarabine, cytarabine, and mitoxantrone; CR1, first complete remission.

a Three or more unrelated chromosomal abnormalities in the absence of 1 of the World Health Organization (WHO)‐designated recurring translocations or inversion.

### 3.2 NGS results

**Figure 1:**
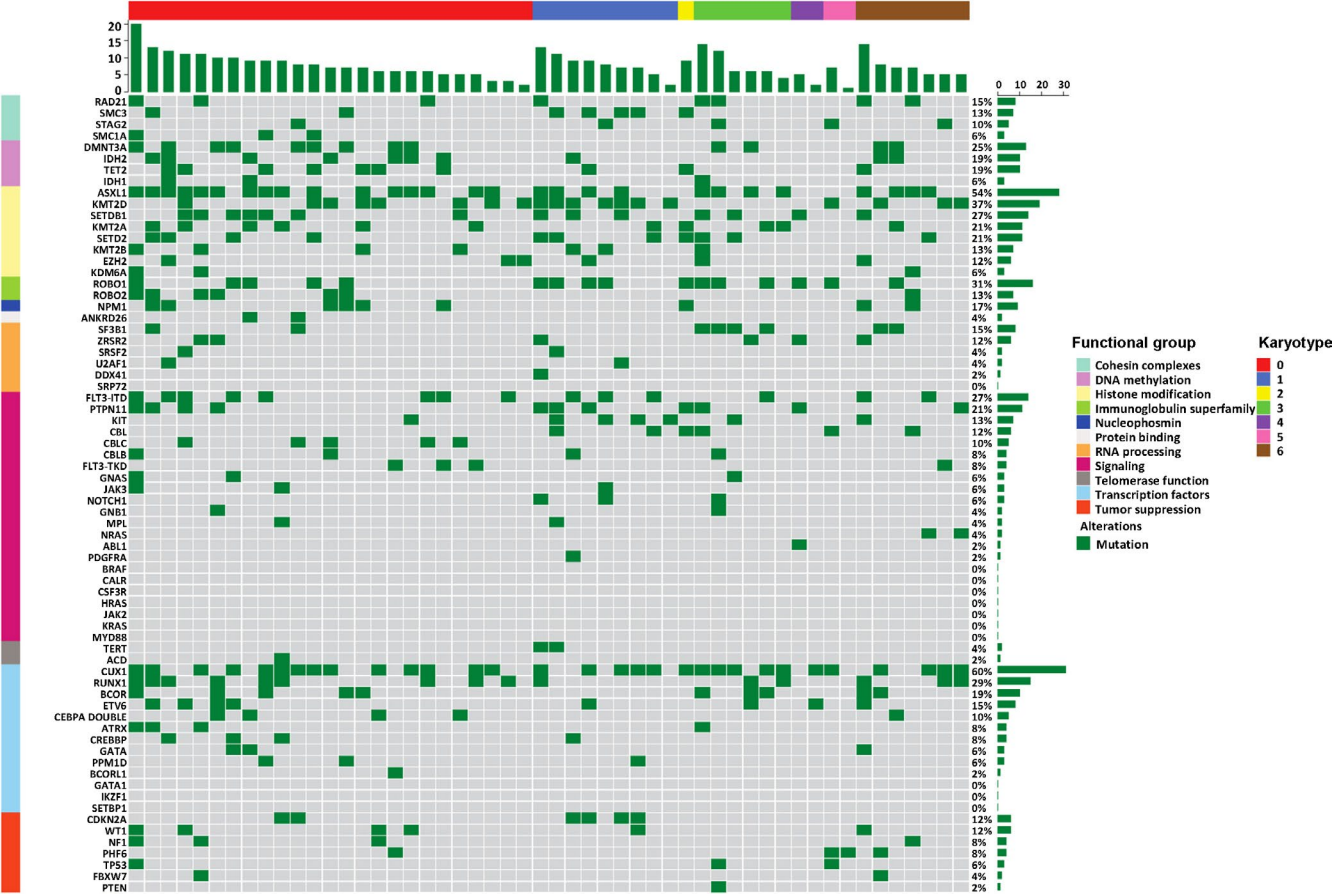
Heatmap shows gene mutations at diagnosis in various functional groups in 52 patients with relapsed/refractory acute myeloid leukemia treated with CLAM. Each small square denotes 1 patient. Karyotype: 0 = normal; 1 = core‐binding factor AML; 2 = t(9;11)(p21.2;q23.3); 3 = inv(3)(q21.3q26.2) or t(3;3)(q21.2;q23.3); 4 = t(v;11q23.3) or del(11)(q23); 5 = complex; 6 = others. AML, acute myeloid leukemia; CLAM, clofarabine, cytarabine, and mitoxantrone

Mutations in at least 1 of the 69 targeted genes were detected in each of the 52 patients (Figure 1) (Files [Link], [Link], [Link]). A median of seven (1‐20) mutations was detected per case. The most frequently mutated genes, classified by putative functions, were those involved in histone modification (N = 48, 92%), gene transcription (N = 44, 85%), cellular signaling (N = 36, 69%), and DNA methylation (N = 26, 50%) (Files S4 and S5). No specific patterns of concurrent mutations could be identified (File S6).

### 3.3 Treatment outcome

All patients completed the first cycle of CLAM (Table 2). The ORR was 90.4% (CR: N = 36, 69.2%; CRi: N = 11, 21.2%). Subsequent to CR/CRi, CLAM consolidation was given to 27 patients (51.9%) (1 cycle, N = 20; 2 cycles, N = 7) and not given to 20 patients because of allogeneic HSCT (N = 9), relapse (N = 9) and unresolved toxicity (persistent thrombocytopenia, N = 1; invasive aspergillosis, N = 1). The median DOR of all responding patients was 5 (1‐26) months, being 5.5 (1‐26) months for CR patients, comparable with that of 4 (2‐23) months for CRi patients (*P* = .67). For CR/CRi patients undergoing allogeneic HSCT, the median DOR was 5.5 (1‐12) months, again comparable with that of 5 (1‐26) months for those not undergoing allogeneic HSCT (*P* = .49).

**Table 2:** Treatment and outcome of 52 patients with acute myeloid leukemia reinduced with CLAM

Parameter	Value
Response	
CR	36 (69.2%)
CRi	11 (21.2%)
Nonremission	5 (9.6%)
CLAM consolidation	
One cycle	20 (38.4%)
Two cycles	7 (13.4%)
Median number of cycles of CLAM consolidation (range)	1 (0‐2)
Allogeneic HSCT^a^	
HLA‐matched sibling donors	13 (25%)
Voluntary‐unrelated donors	11 (21.1%)
Myeloablative conditioning	17 (32.6%)
Reduced‐intensity conditioning	7 (13.4%)
Relapses	21 (40.4%)
Deaths	17 (32.7%)
Deaths from refractory leukemia	16 (30.8%)
Deaths from graft vs host disease	1 (2%)
Deaths within 60 d of the first cycle of CLAM	0
Survivals	
Median overall survival, mo (95% CI)	Not reached
2‐y overall survival	65.8%
Median relapse‐free survival, mo (95% CI)	23 (17‐26)
2‐y relapse‐free survival	45.7%
Median event‐free survival, mo (95% CI)	22 (14‐30)
2‐y event‐free survival	40.2%

Abbreviations: AML, acute myeloid leukemia; CI, confidence interval; CLAM, clofarabine, cytarabine, and mitoxantrone; CR, complete remission; Cri, complete remission with incomplete hematological recovery; HSCT, hematopoietic stem cell transplantation.

a 22 patients received allogeneic HSCT after CLAM‐induced CR/CRi; 1 patient relapsed after CLAM, received salvage azacitidine and decitabine, and then an allogeneic HSCT; 1 patient not responding to CLAM was treated with decitabine and then received an allogeneic HSCT.

### 3.4 Toxicity

Treatment toxicities during CLAM induction and consolidation are shown in Table 3. Grade 3 neutropenia and thrombocytopenia were seen in all cases. Hepatotoxicity manifesting as transaminitis was seen in 31 patients (grade 1‐2, N = 29; grade 3, N = 2). In all cases, transaminases completely normalized within 2 weeks and did not require therapy cessation. Grade 1‐2 rashes occurred in 6 patients and were self‐remitting. Grade 3 febrile neutropenia occurred in all patients requiring empirical antibiotics. Gram‐negative sepsis occurred in 14 patients. Breakthrough invasive fungal diseases during echinocandin prophylaxis occurred in 2 patients (invasive aspergillosis; *Saprochaete* fungemia). No breakthrough invasive fungal disease occurred in patients receiving posaconazole/voriconazole prophylaxis. All patients responded to antimicrobial therapy without life‐threatening complications or hemodynamic disturbances necessitating inotropic or vasopressor support. There were no admissions to the intensive care unit and no treatment‐related mortality (TRM).

**Table 3:** Treatment toxicities of 52 patients during CLAM reinduction and consolidation

Toxicity	Grade 1‐2	Percentage	Grade 3	Percentage
	Number of patients		Number of patients	
Hematologic				
Anemia	29	55.8%	23	44.2%
Neutropenia	0	0	52	100%
Thrombocytopenia	0	0	52	100%
Febrile neutropenia	0	0	52	100%
Nonhematological				
Transaminitis	29	55.7%	2^a^	3.8%
Skin	6	11.5%	0	0
Nausea and vomiting	5	9.6%	0	0
Diarrhea	12	23.1%	0	0
Stomatitis	17	32.7%	0	0

Note

Transaminitis: elevation of alanine aminotransferase and/or aspartate aminotransferase.

Abbreviations: CLAM, clofarabine, cytarabine, and mitoxantrone.

a Grade 3 only.

### 3.5 Prognostic factors for response

Univariate analysis showed that inferior responses (not achieving CR/CRi) were associated with age 18‐45 years (*P* = .01), *CBL* mutations (*P* = .04), and *PPM1D* mutations (*P* = .004) (File S7). On multivariate analysis, none of these parameters were significant. Notably, responses were unaffected by adverse karyotypic aberrations (CR/CRi for t(3;3) or inv(3): 5/6, 83%) and unfavorable gene mutations (CR/CRi for mutated cases: *ASXL1*, 25/28, 89%; *RUNX1*, 14/15, 93%; *FLT3*‐ITD, 14/14, 100%; *IDH2*, 9/10, 90%; *TP53*, 2/3, 67%; *IDH1*, 3/3, 100%) (breakdown of responses according to other karyotypic aberrations and gene mutations can be found in File S7).

### 3.6 Outcome of patients undergoing allogeneic HSCT after CR/Cri

**Figure 2:**
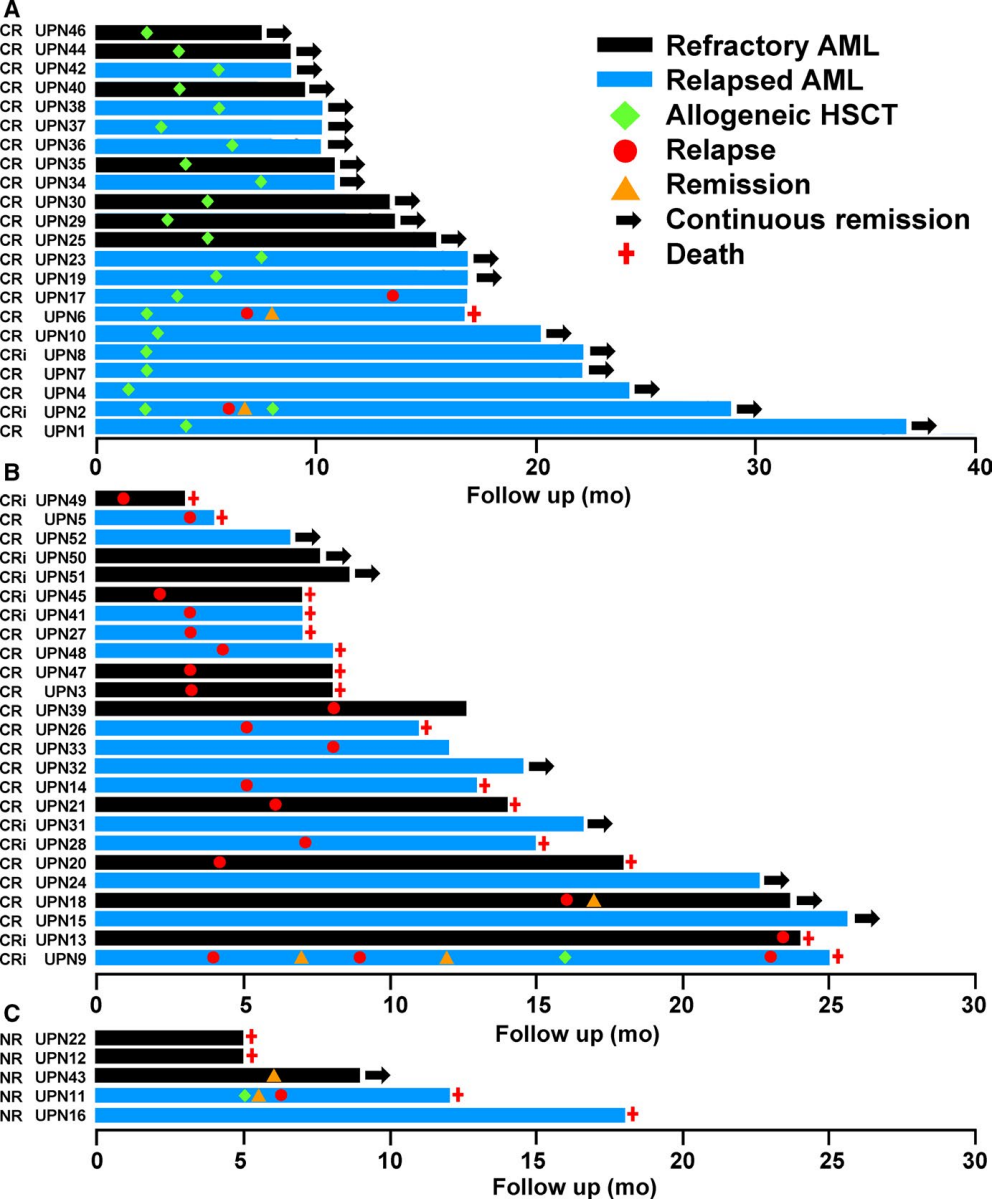
Swimmer plot illustrating the outcome of 52 patients treated with CLAM. A, Patients undergoing allogeneic hematopoietic stem cell transplantation (HSCT) after initially achieving complete remission (CR) or CR with incomplete hematological recovery (CRi) with CLAM. B, Patients who did not undergo allogeneic HSCT after initially achieving CR/CRi with CLAM. C, Patients with nonremission (NR) to CLAM. CLAM, clofarabine, cytarabine, and mitoxantrone

Twenty‐two CR/CRi patients underwent allogeneic HSCT (matched‐sibling donors, N = 11; volunteer‐unrelated donors, N = 11), 9 directly after CLAM reinduction, and 13 after CLAM consolidation (1 cycle, N = 8; 2 cycles, N = 5) (myeloablative conditioning, N = 15; reduced‐intensity conditioning, N = 7) (Figure 2A) (File S8). The median time to engraftment was 15 (10‐21) days. There was no TRM. Acute and chronic GVHD occurred in 11 patients and 7 patients, respectively (other complications were presented in File S8). After a median follow‐up of 21.5 (11‐36) months post‐HSCT, there were three relapses (UPNs 2, 6, and 17) (Figure 2A). UPN2 achieved CR2 with salvage chemotherapy and underwent a second HLA‐matched sibling HSCT, and has remained in continuous remission. UPN6 relapsed as myeloid sarcoma, which responded completely to radiotherapy and donor lymphocyte infusion. However, he developed severe chronic GVHD with bronchiolitis obliterans and died from respiratory failure. UPN17 was refractory to salvage chemotherapy and has remained alive on palliative care.

### 3.7 Outcome of patients not undergoing allogeneic HSCT after CR/Cri

Twenty‐five patients did not receive allogeneic HSCT after CR/CRi (no donor, N = 8; patient refusal, N = 8; relapse, N = 7; HSCT pending, N = 2). After a median follow‐up of 13 (4‐27) months post‐CR/CRi, 7 patients had remained in continuous CR (Figure 2B), whereas 18 patients had relapsed (after CLAM, N = 9; after the first consolidation, N = 8; after second consolidation, N = 1). Relapsed patients had a dismal outcome, with only 2 cases (UPN9 and UPN18) responding to salvage treatment (Figure 2B; File S9). UPN9 relapsed 3 months after CLAM, achieved CRi with azacitidine, relapsed again, achieved CRi with decitabine, underwent an HLA‐matched sibling HSCT, relapsed again, and died from refractory AML. UPN18 relapsed 16 months after CLAM, achieved CRi again with CLAM, and an allogeneic HSCT has been scheduled. For the other relapsed cases, 15 patients had died from refractory leukemia, and 1 patient (UPN33) has remained alive on palliative care.

### 3.8 Outcome of NR patients

Five patients did not respond to CLAM (Figure 2C; File S9). Three patients were refractory to other salvage regimens and died of refractory leukemia. One patient (UPN11) responded to decitabine salvage, underwent a matched‐sibling HSCT, but relapsed 2 months afterward and died from refractory leukemia. One patient (UPN43) achieved CRi after decitabine salvage, with HSCT pending.

### 3.9 Survivals and prognostic factors

**Figure 3:**
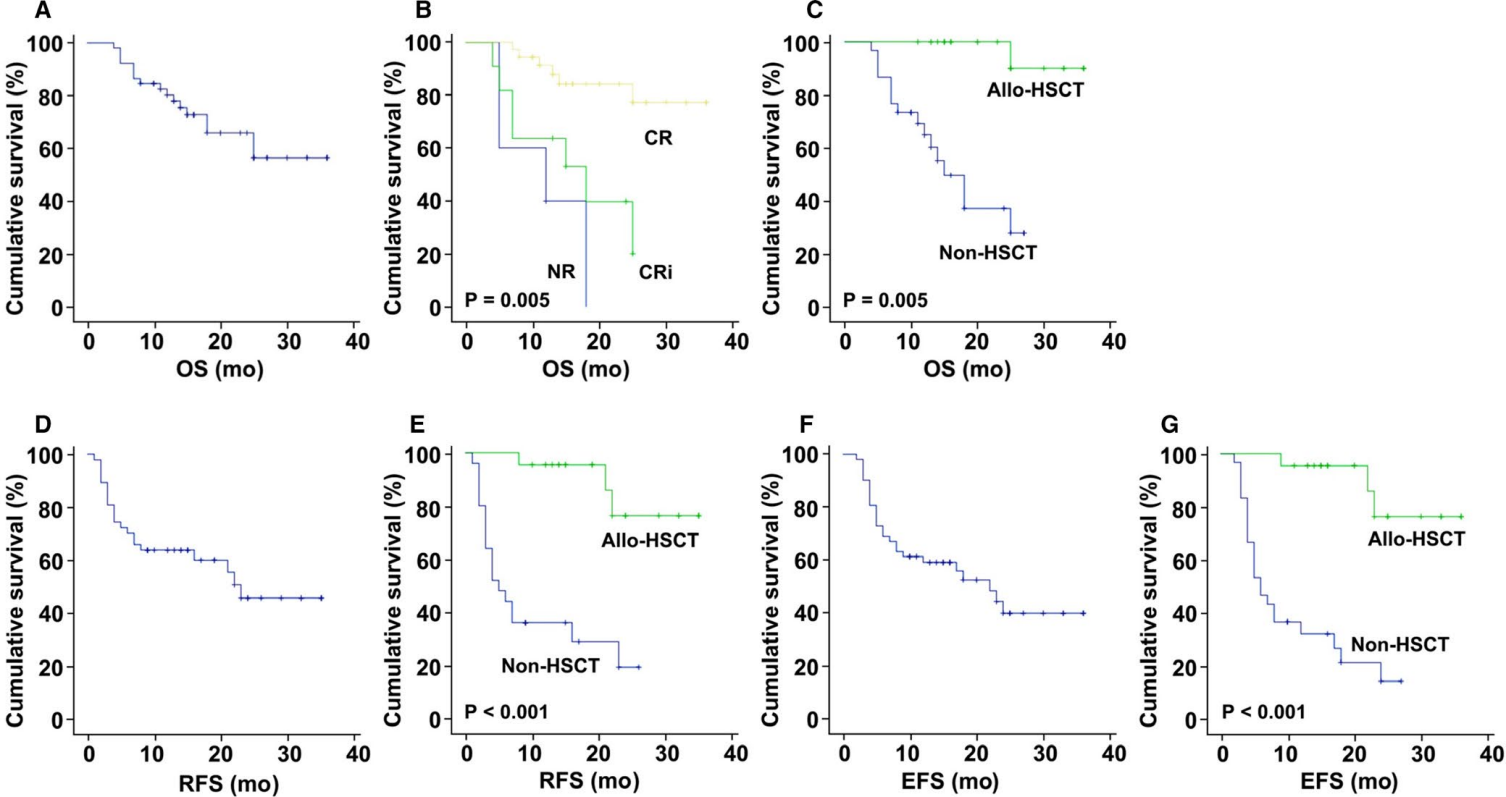
Survivals and prognostic factors for survivals in 52 patients with refractory/relapsed acute myeloid leukemia treated with CLAM. A‐C, overall survival (OS); D and E, relapse‐free survival (RFS); F and G, event‐free survival (EFS). CLAM, clofarabine, cytarabine, and mitoxantrone

The 2‐year OS, RFS, and EFS were 65.8%, 45.7%, and 40.2%, respectively (Figure 3). Prognostic factors for OS, RFS, and EFS were determined (Table 4; File S10). For OS, univariate analysis showed that significantly superior survivals were associated with CR after CLAM (*P* = .005) and allogeneic HSCT (*P* = .005). Multivariate analysis showed that superior OS was still associated with CR after CLAM (*P* = .02) and allogeneic HSCT (*P* = .02). For RFS, univariate analysis showed that superior survivals were associated with *CUX1* mutation (*P* = .03), CR after CLAM (*P* = .008), and allogeneic HSCT (*P* < .001). On multivariate analysis, superior RFS was only associated with allogeneic HSCT (*P* = .003). For EFS, univariate analysis showed that significantly superior survivals were associated with CR after CLAM (*P* = .01) and allogeneic HSCT (*P* < .001). On multivariate analysis, superior EFS was only associated with allogeneic HSCT (*P* = .003). It is noteworthy that clinicopathologic features, karyotypic aberrations, and mutations of genes known to have unfavorable impacts (*ASXL1*, *DNMT3A*, *FLT3*‐ITD, *IDH1*, *IDH2*, *KMT2A*, *RUNX1*, and *TP53*) were not different between patients receiving and not receiving allogeneic HSCT (File S11). Hence, the only two factors significantly associated with survivals on multivariate analysis were CR after CLAM (with OS) and allogeneic HSCT (with OS, RFS, and EFS). Remarkably, patients achieving CR after CLAM had a 2‐year OS of 84.3%, and patients receiving allogeneic HSCT had 2‐year OS of 90%, and RFS and EFS both of 76.4%.

**Table 4:** Prognostic factors for survivals in 52 patients treated with CLAM

Parameter	No.	Overall survival			Relapse‐free survival			Event‐free survival		
		2‐y (%)	HR (95% CI)	*P* value	2‐y (%)	HR (95% CI)	*P* value	2‐y (%)	HR (95% CI)	*P* value
Gender										
Male	26	64.5	0.83 (0.32‐2.17)	.71	43.8	0.80 (0.34‐1.91)	.62	38.3	0.78 (0.36‐1.71)	.54
Female	26	61.8	1.20 (0.46‐3.14)		39.9	1.25 (0.53‐2.97)		34.9	1.28 (0.59‐2.79)	
Age										
18‐45	24	56.0	1.43 (0.55‐3.72)	.46	25.6	1.42 (0.60‐3.34)	.43	17.8	1.74 (0.80‐3.80)	.17
46‐65	28	66.6	0.70 (0.27‐1.81)		51.3	0.71 (0.30‐1.67)		51.8	0.58 (0.26‐1.26)	
Status										
Relapse	31	62.3	1.28 (0.48‐3.41)	.62	51.7	0.68 (0.29‐1.62)	.39	48.3	0.71 (0.33‐1.53)	.38
Refractory	21	64.4	0.78 (0.29‐2.07)		28.7	1.47 (0.62‐3.46)		23.7	1.42 (0.65‐3.08)	
Response										
CR	36	84.3	0.11 (0.03‐0.40)	.005	48.5	0.28 (0.11‐0.72)	.008	48.5	0.24 (0.08‐0.71)	.01
CRi	11	39.8	4.81 (1.60‐14.42)		18.2	3.57 (1.40‐9.14)		18.2	3.95 (1.54‐10.13)	
NR	5	0	9.32 (2.51‐34.55)		—	—		0	4.18 (1.42‐12.30)	
Allogeneic HSCT										
Performed^a^	22	90.0	0.05 (0.007‐0.42)	.005	76.4	0.13 (0.04‐0.40)	<.001	76.4	0.15 (0.05‐0.39)	<.001
Not performed	30	37.3	18.35 (2.40‐140.37)		19.2	7.59 (2.50‐23.04)		14.3	6.90 (2.55‐18.67)	

Abbreviations: AML, acute myeloid leukemia; Ara‐C, mitoxantrone; CI, confidence interval; CLAM, clofarabine, cytarabine, and mitoxantrone; CR, complete remission; Cri, complete remission with incomplete hematological recovery; EFS, event‐free survival; HSCT, hematopoietic stem cell transplantation; HR, hazard ratio for event; NR, nonremission; OS, overall survival; R1, first relapse; RFS, relapse‐free survival.

a 2 patients who did not undergo HSCT initially, but underwent HSCT following response to salvage treatment, belonged to the non‐HSCT group.

## 4 DISCUSSION

Clofarabine‐containing regimens had previously been tested in refractory/relapsed AML. Most combinations contained clofarabine and cytarabine, with ORR (CR) ranging from 38% (22%),^9^ 47% (35%),^18^ to 61% (46%).^17^ Results were not improved by adding idarubicin to clofarabine/cytarabine (ORR: 38%).^36^ Combining clofarabine with etoposide and mitoxantrone led to a comparable ORR of 36%.^27^ Our previous study of clofarabine/cytarabine showed a similar ORR of 43%.^16^ Because clofarabine is lymphoablative, clofarabine/cytarabine suppresses both myeloid and lymphoid cells, increasing the susceptibility to bacterial, fungal, and viral infections.^16^, ^27^ In designing CLAM, we reduced the dose of clofarabine and cytarabine, with the objective of decreasing infective risks to facilitate subsequent allogeneic HSCT. To maintain efficacy we added mitoxantrone, which is active in refractory AML.^2^ Surmising CLAM to be safer but perhaps not more effective than clofarabine/cytarabine, we powered the study to only show a ≥20% improvement in ORR over conventional regimens. As anticipated, CLAM was safe with few infective complications. Unexpectedly, however, CLAM was much more active, showing an ORR of 90.4% and CR of 69.2%. These results were apparently superior to those of other clofarabine/cytarabine^9^, ^17^, ^18^ and clofarabine/mitoxantrone^27^ combinations. The reasons why our results are apparently better are not clear. We recruited patients failing only one prior regimen, who might not be as heavily pretreated as those in other studies, which considered cases failing two or more regimens as refractory patients. This approach limited cumulative chemotherapy‐related organ toxicities in potential HSCT candidates. Our patients were also younger with a median age at 46 years and had good performance status. Furthermore, we followed the proposed synergistic interaction between fludarabine and cytarabine^44^ in administering cytarabine exactly 4 hours after clofarabine. Such synergism might be further enhanced by mitoxantrone. Hence, more mechanistic investigations are needed.

Our cohort comprised high‐risk karyotypes and genetic mutations, which might have accounted for the original refractoriness or relapse following “3 + 7” induction. Rearrangement of 3q21q26, occurring otherwise in 1% of AML,^40^ was found in 11% of our cases. Mutations of genes with adverse prognostic impact were also more frequent in our cohort as compared with unselected cases of AML, including *ASXL1* (54% vs 5‐10%), *RUNX1* (29% vs 5‐10%), *KMT2A* (27% vs 5%), *TET2* (19% vs 5‐10%), and *KIT* (13% vs <5%).^40^, ^45^ Other gene mutations with adverse impacts were found at frequencies comparable with unselected AML, including *FLT3* (33%) and *TP53* (6%). Despite that, treatment outcome was apparently unaffected.

Current strategies targeting gene mutations have shown favorable responses, including quizartinib for *FLT3*‐mutants (ORR: 50%, CR: 3%),^46^ ivosidenib for *IDH1*‐mutants (ORR, 41.6%; CR: 21.6%),^47^ enasidenib for *IDH2*‐mutants (ORR: 38.8%, CR: 19.6%),^48^ and decitabine for *TP53*‐mutants (ORR: 37.5‐100%).^49^, ^50^ With all the limitations of comparing different studies, including disparate designs and patients populations, the efficacy of CLAM (*FLT3*‐mutants, ORR: 100%, CR: 72%; *IDH1*‐mutants, ORR: 100%, CR: 0%; *IDH2*‐mutants, ORR: 90%, CR: 60%; *TP53*‐mutants, ORR: 67%, CR: 33%) compared favorably with the results achieved by gene‐targeting approaches.

The main toxicity of CLAM was hematologic and all cases developed grade 3 neutropenia, thrombocytopenia, and neutropenic fever requiring empirical antibiotics. However, with vigorous supportive care and G‐CSF administration, none of these patients developed serious infective complications. For HSCT‐eligible patients, none had CLAM‐related complications that hindered transplantation. Furthermore, during and after allogeneic HSCT, there were no TRM and other untoward complications attributable to CLAM.

Two factors were important for OS. Patients achieving CR had a superior 2‐year OS of 84.3%, indicating that the quality of remission was relevant. Patients undergoing allogeneic HSCT had a more impressive 2‐year OS of 90%. Allogeneic HSCT was the only factor that was important for RFS and EFS, with 76.4% of these patients surviving without disease at 2 years. Remarkably, for 22 patients who underwent allogeneic HSCT after CLAM‐induced CR/CRi, 20 patients have still remained in continuous remission. These results showed that CLAM was highly effective in bridging refractory/relapsed patients to allogeneic HSCT and this was achieved without significant toxicity that compromised the procedure. It is notable that even for patients not undergoing allogeneic HSCT after CR/CRi, 7 of 25 patients were still in remission, with 4 patients already having a median follow‐up of about 18 months (Figure 2B).

In conclusion, CLAM was highly effective for refractory/relapsed AML, with its efficacy not apparently affected by high‐risk karyotypes and genetic mutations. Toxicity was manageable and did not compromise subsequent allogeneic HSCT, which when performed after CLAM‐induced CR/CRi resulted in excellent survivals. Treatment of refractory/relapsed AML has become more oriented toward targeting of individual gene mutations. In this era of molecular targeting, CLAM might still have a role to play. It offers the advantage of a highly effective regimen that is readily available. It provides a median DOR of 5 months, which is meaningful for organization of HSCT. Delays associated with recruitment into clinical trials or sourcing of targeted drugs are obviated. Precious time is saved, so that patients can quickly be bridged to a potentially curative allogeneic HSCT.

## Data Availability

We agree to share data when this is applicable.
