# Supplementary material for: Clofarabine, cytarabine, and mitoxantrone in refractory/relapsed acute myeloid leukemia: High response rates and effective bridge to allogeneic hematopoietic stem cell transplantation
Source: Cancer Med. 2020 Mar 18;9(10):3371–82. doi: 10.1002/cam4.2865 (PMC7221314; doi:10.1002/cam4.2865)
Supplement: Supplementary file 1 [file CAM4-9-3371-s001.doc]

| **No. 88**  **K6N Tel: 6079, Fax: 3960** |  | **Date : _____________________________ Course : _____________________________**  **Dose adjustment: ____________________________**  **Ht.(cm):_________ Wt.(kg):_______ SA(m^2^):______**  **Drug Allergy : ⬜ No known drug allergy**  **⬜ Yes, please specify: ___________**  **Hepatitis status:**  **HBsAg positivity: ⬜ Yes ⬜ No**  **Antiviral prophylaxis if positive: ⬜ Yes ⬜ No** |
| --- | --- | --- |
| **Patient’s gum label** |  |

***Chemotherapy: CLAM: Clofarabine(Evoltra ®), Cytarabine (Ara-C), Mitoxantrone for AML***

***For Compassionate Use ONLY* 🞎**

**Clofarabine** (30mg/m^2^/day) into 100/ 150/ 200ml NS over 1 hour from **Day 1 to Day 5**

**(Evoltra ®) **Administer with 0.2 micrometer in-line filter***

|  | **Cytarabine (Ara-C)** | | | (750mg/m^2^/day) | | into 500ml NS over 2 hours from **Day 1 to Day 5**.  ✽(4 hour after clofarabine) | | | | |  |
| --- | --- | --- | --- | --- | --- | --- | --- | --- | --- | --- | --- |
|  | **Mitoxantrone** | | | (12mg/m^2^/day) | | into 100ml NS over 1 hour from **Day 3 to Day 5**. | | | | |  |
| ***Day*** | | ***Date*** | ***Route/Drug*** | | ***Dose*** | | ***Adminstration***  ***(Solution/ duration)*** | ***Clinician’s signature*** | ***Pharmacist’s signature*** | ***(Time)Drug given by*** | |
| D1 | | ____ | IV Clofarabine  IV Cytarabine  (4 hour after clofarabine) | | ____mg  ____mg | | into 100/ 150/ 200ml NS over 1 hr  into 500ml NS over 2 hr | ________  ________ | _________  _________ | (___)  (___) | |
| D2 | | ____ | IV Clofarabine  IV Cytarabine  (4 hour after clofarabine) | | ____mg  ____mg | | into 100/ 150/ 200ml NS over 1 hr  into 500ml NS over 2 hr | ________  ________ | _________  _________ | (___)  (___) | |
| D3 | | ____ | IV Clofarabine  IV Cytarabine  (4 hour after clofarabine)  IV Mitoxantrone | | ____mg  ____mg  ____mg | | into 100/ 150/ 200ml NS over 1 hr  into 500ml NS over 2 hr  into 100ml NS over 1 hr | ________  ________  ________ | _________  _________  _________ | (___)  (___)  (___) | |
| D4 | | ____ | IV Clofarabine  IV Cytarabine  (4 hour after clofarabine)  IV Mitoxantrone | | ____mg  ____mg  ____mg | | into 100/ 150/ 200ml NS over 1 hr  into 500ml NS over 2 hr  into 100ml NS over 1 hr | ________  ________  ________ | _________  _________  _________ | (___)  (___)  (___) | |
| D5 | | ____ | IV Clofarabine  IV Cytarabine  (4 hour after clofarabine)  IV Mitoxantrone | | ____mg  ____mg  ____mg | | into 100/ 150/ 200ml NS over 1 hr  into 500ml NS over 2 hr  into 100ml NS over 1 hr | ________  ________  ________ | _________  _________  _________ | (___)  (___)  (___) | |

*****See instruction on next page**

**(Designated Physician: Prof. Kwong Yok Lam, Dr. Harry Gill)** Endorsement by clinician: _______________

***Instruction of Chemotherapy: CLAM: Clofarabine(Evoltra ®), Cytarabine (Ara-C), Mitoxantrone for AML***

**Accompanying medications:**

- With antiemetic: a 5HT3 antagonist Intravenous Ondansetron (Zofran) 8mg Q12H Day 1 to Day 5and

NK-1 receptor antagonist.

- Decadron (Dexamethasone 0.1%) eye drops LA Both Eye (BE) Q4H from Day 1 to Day 5.

**Remarks and Precautions:**

1. Repeat echocardiogram before CLAM
2. Recommended dilution of clofarabine:

| Surface area (per m^2^) | Total diluted volume of clofarabine |
| --- | --- |
| ≤ 1.44 | 100ml NS |
| 1.45 – 2.4 | 150ml NS |
| 2.41 to 2.5 | 200ml NS |

1. Administer with 0.2 micrometer in-line filter for clofarabine administration
2. Prophylaxis of Pneumocystis jirovecii pneumonia (PJP) with oral co-trimoxazole (Septrin) or inhaled Pentamidine.
3. Antifungal prophylaxis with oral posaconazole 200mg tds or syrup itraconazole 200mg daily.
4. In case of deranged LFT or if there are concerns about absorption such as during severe mucositis or diarrhea, use intravenious echinocandin (micafungin or anidulafungin) as antifungal prophylaxis.
5. Daily monitoring for CBC,L/RFT, RG, CaPO_4_, LDH, urate.
6. Weekly ECG and Chest X-ray monitoring and whenever necessary.
7. Daily G-CSF support to keep absolute neutrophil count above 1 x 10^9^/L starting Day 6.
8. Packed red cell and platelet support as necessary.

- Medications will not be dispensed if hepatitis status is not filled in (except for the first cycle).

**References:**

1. Faderl S, Ferrajoli A, Wierda W, et al. Clofarabine combinations as acute myeloid leukemia salvage therapy. *Cancer.* Oct 15 2008;113(8):2090-2096.
2. Becker PS, Kantarjian HM, Appelbaum FR, et al. Clofarabine with high dose cytarabine and granulocyte colony-stimulating factor (G-CSF) priming for relapsed and refractory acute myeloid leukaemia. *Br J Haematol.* Oct 2011;155(2):182-189.
3. Tse E, Leung AY, Sim J, et al. Clofarabine and high-dose cytosine arabinoside in the treatment of refractory or relapsed acute myeloid leukaemia. *Ann Hematol.* Nov 2011;90(11):1277-1281.
4. Nazha A, Kantarjian H, Ravandi F, et al. Clofarabine, idarubicin, and cytarabine (CIA) as frontline therapy for patients </=60 years with newly diagnosed acute myeloid leukemia. *Am J Hematol.* Nov 2013;88(11):961-966.
5. Vigil CE, Tan W, Deeb G, et al. Phase II trial of clofarabine and daunorubicin as induction therapy for acute myeloid leukemia patients greater than or equal to 60 years of age. *Leuk Res.* Nov 2013;37(11):1468-1471.
