# Supplementary material for: Clofarabine, cytarabine, and mitoxantrone in refractory/relapsed acute myeloid leukemia: High response rates and effective bridge to allogeneic hematopoietic stem cell transplantation
Source: Cancer Med. 2020 Mar 18;9(10):3371–82. doi: 10.1002/cam4.2865 (PMC7221314; doi:10.1002/cam4.2865)
Supplement: Supplementary file 2 [file CAM4-9-3371-s002.docx]

**Supplemental file 2. Methodology for next generation sequencing.**

Targeted NGS was performed on DNA samples from diagnostic bone marrow aspirates in all cases. A custom xGen Lockdown Panel targeting 69 genes was designed based on GRCh37/hg19 (Integrated DNA Technologies, Coralville, Iowa, USA). The targeted genes included *ABL1, ACD, ANKRD26, ASXL1, ATRX, BCOR, BCORL1, BRAF, CALR, CBL, CBLB, CBLC, CDKN2A, CEBPA, CREBBP, CSF3R, CUX1, DDX41, DNMT3A, ETV6, EZH2, FBXW7, FLT3, GATA1, GATA2, GNAS, GNB1, HRAS, IDH1, IDH2, IKZF1, JAK2, JAK3, KDM6A, KIT, KMT2A, KMT2B, KMT2D, KRAS, MPL, MYD88, NF1, NOTCH1, NPM1, NRAS, PDGFRA, PHF6, PPM1D, PTEN, PTPN11, RAD21, ROBO1, ROBO2, RUNX1, SETBP1, SETD2, SETDB1, SF3B1, SMC1A, SMC3, SRP72, SRSF2, STAG2, TERT, TET2, TP53, U2AF1, WT1,* and *ZRSR2.* All exons of the 69 genes were sequenced, with a total of 2885 probes covering 273.03 kb. The enriched libraries were sequenced pair-ended with the Illumina MiSeq System (Illumina, San Diego, California, USA). FASTQ files containing at least 1 million raw reads with coverage of 500X were generated for subsequent bioinformatic analyses as previously described.[^1^](#_ENREF_1) Briefly, single nucleotide variants (SNV) and insertions/deletions (INDELs) were called independently by Mutect2 and VarScan.[^1^](#_ENREF_1) Mutect2 somatic calling incorporated normal controls prepared from 4 bone marrow samples and 2 peripheral blood samples from 6 healthy unrelated donors sequenced in parallel. VarScan calling incorporated the same normal controls to exclude non-tumour-specific variants. The resulting variants called by Mutect2 and VarScan were concatenated. For detection of *FLT3* internal tandem duplication (*FLT3-*ITD), Pindel^[1](#_ENREF_1)^ was applied and subsequently filtered with normal controls similarly. The resulting variants were annotated by ANNOVAR[^1^](#_ENREF_1) and SNPeff [^2^](#_ENREF_2), using the public databases including esp6500siv2 ([http://evs.gs.washington.edu/EVS/](http://evs.gs.washington.edu/EVS/#_blank)), 1000g2015aug[^3^](#_ENREF_3), clinvar_20180603[^4^](#_ENREF_4), dbnsfp33a[^5^](#_ENREF_5), cosmic70[^6^](#_ENREF_6), gnomAD^[7](#_ENREF_7)^ and TCGA[^8^](#_ENREF_8). All variants detected were processed with the variant frequency filter (< 1% in 1000 genomes), allele frequency filter (> 5%) and coverage filter (> 500 x). The filtered variants were then annotated based on variant prediction (variant type, Polyphen2, SIFT, LRT, Mutation Taster) and actionability (genes or variants with prognostic and therapeutic significance) and predicted as mutations.[^9^](#_ENREF_9) Graphs and charts were constructed using Graphpad Prism version 7.02 and R software (The R Project for Statistical Computing). Gene association and correlation was performed by GeneNet V1.2.13 (R package) with Graphical Gaussian model. Covariance selection was used to determine gene associations. Concentration graph analysis was used to determine the gene relevance network, generating a covariance matrix for Circos plot (Circos software).

**References:**

1. Gill H, Ip HW, Yim R, et al. Next-generation sequencing with a 54-gene panel identified unique mutational profile and prognostic markers in Chinese patients with myelofibrosis. *Ann Hematol*. 2018.

2. Cingolani P, Platts A, Wang le L, et al. A program for annotating and predicting the effects of single nucleotide polymorphisms, SnpEff: SNPs in the genome of Drosophila melanogaster strain w1118; iso-2; iso-3. *Fly (Austin)*. 2012;6(2):80-92.

3. The Genomes Project C, Auton A, Abecasis GR, et al. A global reference for human genetic variation. *Nature*. 2015;526:68.

4. Landrum MJ, Lee JM, Benson M, et al. ClinVar: improving access to variant interpretations and supporting evidence. *Nucleic Acids Res*. 2018;46(D1):D1062-d1067.

5. Liu X, Jian X, Boerwinkle E. dbNSFP: A lightweight database of human nonsynonymous SNPs and their functional predictions. *Human Mutation*. 2011;32(8):894-899.

6. Tate JG, Bamford S, Jubb HC, et al. COSMIC: the Catalogue Of Somatic Mutations In Cancer. *Nucleic Acids Research*. 2018:gky1015-gky1015.

7. Lek M, Karczewski KJ, Minikel EV, et al. Analysis of protein-coding genetic variation in 60,706 humans. *Nature*. 2016;536:285.

8. Dong C, Wei P, Jian X, et al. Comparison and integration of deleteriousness prediction methods for nonsynonymous SNVs in whole exome sequencing studies. *Human Molecular Genetics*. 2015;24(8):2125-2137.

9. Sukhai MA, Craddock KJ, Thomas M, et al. A classification system for clinical relevance of somatic variants identified in molecular profiling of cancer. *Genet Med*. 2016;18(2):128-136.
