# Supplementary material for: Clofarabine, cytarabine, and mitoxantrone in refractory/relapsed acute myeloid leukemia: High response rates and effective bridge to allogeneic hematopoietic stem cell transplantation
Source: Cancer Med. 2020 Mar 18;9(10):3371–82. doi: 10.1002/cam4.2865 (PMC7221314; doi:10.1002/cam4.2865)
Supplement: Supplementary file 3 [file CAM4-9-3371-s003.docx]

**Supplemental file 3. Karyotypes of 52 patients with relapsed or refractory acute myeloid leukemia.**

| **Karyotype** | **No. of patients** |
| --- | --- |
|  |  |
| Normal | 25 |
| Abnormal | 27 |
| t(8;21)(q22;q22.1) | 2 |
| t(8;21)(q22;q22.1), -Y | 2 |
| t(8;21)(q22;q22.1),t(8;9),-Y | 1 |
| t(8;21)(q22;q22.1),-X | 1 |
| t(8;21)(q22;q22.1),del(7)(q32) | 1 |
| inv(16)(p13.1q22) | 2 |
| t(9;11)(p21.2;q23.3) | 1 |
| del(11)(q23) | 1 |
| del(11)(q23),+8,+21,-X | 1 |
| inv(3)(q21.3q26.2) | 2 |
| inv(3)(q21.3q26.2),+X | 1 |
| inv(3)(q21.3q26.2),-7 | 1 |
| t(3;3)(q21.3;q26.2) | 2 |
| add(1)(p36.3),+8,der(8;17)(q10;q10),del(16)(p13.1) | 1 |
| Hyperdiploidy with hexasomy 21, trisomies 15 and 18 and -Y | 1 |
| Trisomy 13 | 1 |
| 93,XXYY[2]/XY[17] | 1 |
| del(9)(q13q22) | 1 |
| add(9)(p13)/del(9)(q13q22) | 1 |
| inv(11)(q12q24) | 1 |
| Trisomy 19 | 1 |
| t(11;17)(p10;q10) | 1 |
|  |  |
