# Supplementary figures and images for: Clofarabine, cytarabine, and mitoxantrone in refractory/relapsed acute myeloid leukemia: High response rates and effective bridge to allogeneic hematopoietic stem cell transplantation
Source: Cancer Med. 2020 Mar 18;9(10):3371–82. doi: 10.1002/cam4.2865 (PMC7221314; doi:10.1002/cam4.2865)

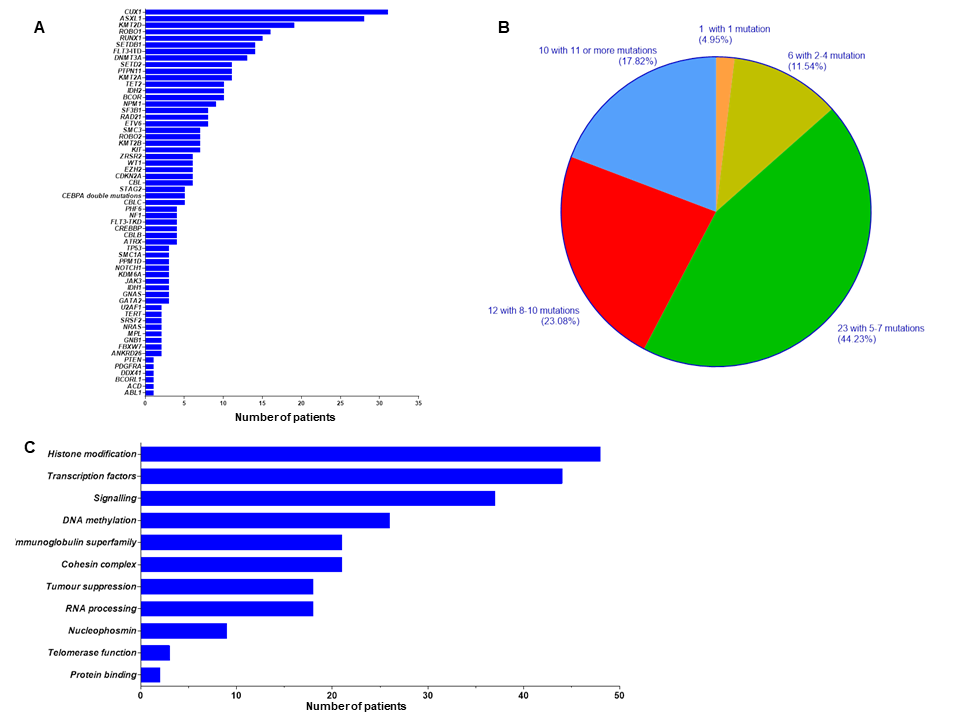

Supplement: Supplementary file 4 [file CAM4-9-3371-s004.TIF]

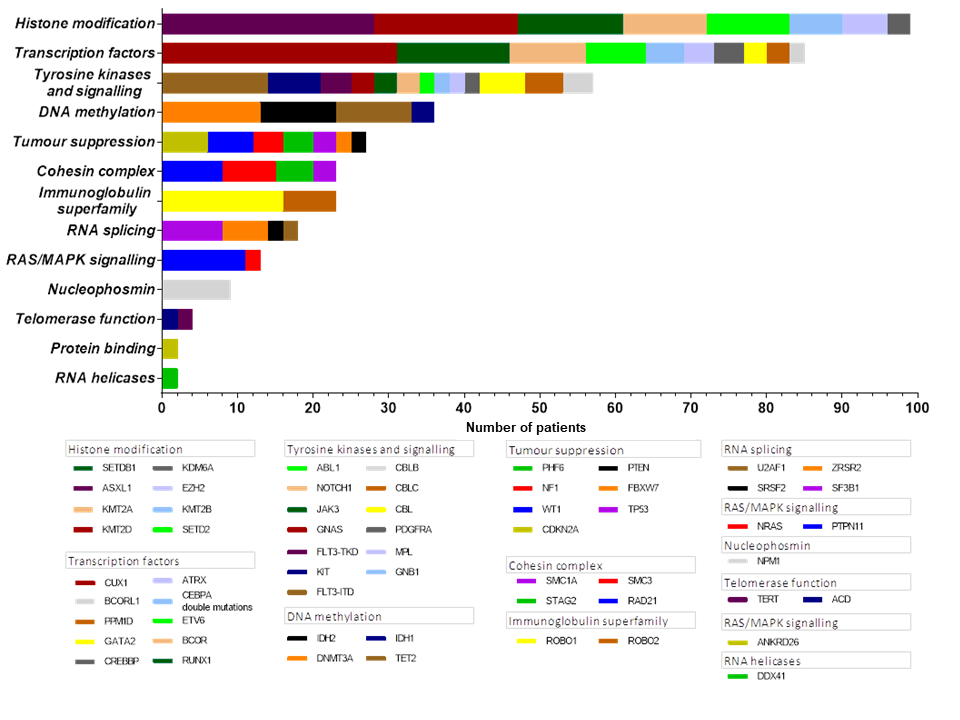

Supplement: Supplementary file 5 [file CAM4-9-3371-s005.TIF]

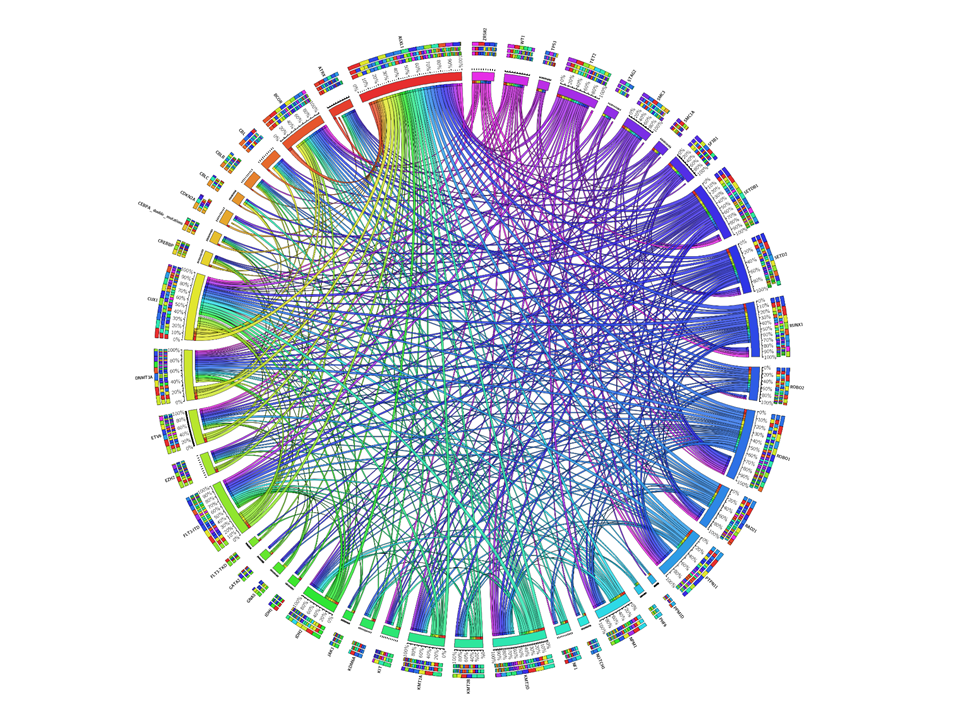

Supplement: Supplementary file 6 [file CAM4-9-3371-s006.TIF]
