# Supplementary material for: Clofarabine, cytarabine, and mitoxantrone in refractory/relapsed acute myeloid leukemia: High response rates and effective bridge to allogeneic hematopoietic stem cell transplantation
Source: Cancer Med. 2020 Mar 18;9(10):3371–82. doi: 10.1002/cam4.2865 (PMC7221314; doi:10.1002/cam4.2865)
Supplement: Supplementary file 7 [file CAM4-9-3371-s007.docx]

**Supplemental file 7. Prognostic factors for response in 52 patients with relapsed or refractory AML treated with CLAM.**

|  | **Number of patients** | |  |
| --- | --- | --- | --- |
| **Parameter** | **CR/CRi** | **NR** | **P-value^#^** |
|  |  |  |  |
| Gender |  |  |  |
| Male | 24 | 2 |  |
| Female | 23 | 3 | 0.64 |
| Age |  |  |  |
| 18-45 years | 19 | 5 |  |
| 46-65 years | 28 | 0 | 0.01 |
| Status prior to CLAM |  |  |  |
| First relapse (R1) | 29 | 2 |  |
| Refractory | 18 | 3 | 0.35 |
| Time to R1 prior to CLAM |  |  |  |
| >12 months from CR1 | 19 | 0 |  |
| ≤12 months from CR1 | 10 | 2 | 0.07 |
| Karyotypes |  |  |  |
| Normal | 25 | 0 |  |
| t(8;21)(q22;q22.1) or inv(16)(p13.1q22)/t(16;16)(p13.1;q22) | 8 | 1 |  |
| t(9;11)(p21.2;q23.3) | 1 | 0 |  |
| inv(3)(q21.3q26.2)/t(3;3)(q21.3;q26.2) | 5 | 1 |  |
| t(v;11q23.3) / del(11)(q23) | 2 | 0 |  |
| Complex | 1 | 1 |  |
| Others | 5 | 2 | 0.13 |
| Gene mutations |  |  |  |
| *CUX1* |  |  |  |
| Mutant | 27 | 4 |  |
| Wild-type | 20 | 1 | 0.33 |
| *ASXL1* |  |  |  |
| Mutant | 25 | 3 |  |
| Wild-type | 22 | 2 | 0.77 |
| *KMT2D* |  |  |  |
| Mutant | 16 | 3 |  |
| Wild-type | 31 | 2 | 0.25 |
| *ROBO1* |  |  |  |
| Mutant | 15 | 1 |  |
| Wild-type | 32 | 4 | 0.58 |
| *RUNX1* |  |  |  |
| Mutant | 14 | 1 |  |
| Wild-type | 33 | 4 | 0.65 |
| *SETDB1* |  |  |  |
| Mutant | 13 | 1 |  |
| Wild-type | 34 | 4 | 0.71 |
| *FLT3* |  |  |  |
| ITD | 14 | 0 |  |
| TKD | 4 | 0 |  |
| Wild-type | 29 | 5 | 0.15 |
| *DNMT3A* |  |  |  |
| Mutant | 12 | 1 |  |
| Wild-type | 35 | 4 | 0.79 |
| *SETD2* |  |  |  |
| Mutant | 11 | 0 |  |
| Wild-type | 36 | 5 | 0.22 |
| *PTPN11* |  |  |  |
| Mutant | 11 | 0 |  |
| Wild-type | 36 | 5 | 0.22 |
| *KMT2A* |  |  |  |
| Mutant | 10 | 1 |  |
| Wild-type | 37 | 4 | 0.95 |
| *TET2* |  |  |  |
| Mutant | 10 | 0 |  |
| Wild-type | 37 | 5 | 0.25 |
| *IDH2* |  |  |  |
| Mutant | 9 | 1 |  |
| Wild-type | 38 | 4 | 0.96 |
| *BCOR* |  |  |  |
| Mutant | 9 | 1 |  |
| Wild-type | 38 | 4 | 0.96 |
| *NPM1* |  |  |  |
| Mutant | 8 | 1 |  |
| Wild-type | 39 | 4 | 0.87 |
| *SF3B1* |  |  |  |
| Mutant | 7 | 1 |  |
| Wild-type | 40 | 4 | 0.76 |
| *RAD21* |  |  |  |
| Mutant | 7 | 1 |  |
| Wild-type | 40 | 4 | 0.76 |
| *ETV6* |  |  |  |
| Mutant | 8 | 0 |  |
| Wild-type | 39 | 5 | 0.32 |
| *SMC3* |  |  |  |
| Mutant | 6 | 1 |  |
| Wild-type | 41 | 4 | 0.65 |
| *ROBO2* |  |  |  |
| Mutant | 6 | 1 |  |
| Wild-type | 41 | 4 | 0.65 |
| *KMT2B* |  |  |  |
| Mutant | 7 | 0 |  |
| Wild-type | 40 | 5 | 0.35 |
| *KIT* |  |  |  |
| Mutant | 7 | 0 |  |
| Wild-type | 40 | 5 | 0.35 |
| *ZRSR2* |  |  |  |
| Mutant | 6 | 0 |  |
| Wild-type | 41 | 5 | 0.40 |
| *WT1* |  |  |  |
| Mutant | 6 | 0 |  |
| Wild-type | 41 | 5 | 0.40 |
| *EZH2* |  |  |  |
| Mutant | 6 | 0 |  |
| Wild-type | 41 | 5 | 0.40 |
| *CDKN2A* |  |  |  |
| Mutant | 5 | 1 |  |
| Wild-type | 42 | 4 | 0.53 |
| *CBL* |  |  |  |
| Mutant | 4 | 2 |  |
| Wild-type | 43 | 3 | 0.04 |
| *STAG2* |  |  |  |
| Mutant | 4 | 1 |  |
| Wild-type | 43 | 4 | 0.41 |
| *CEBPA* |  |  |  |
| Double mutations | 5 | 0 |  |
| Wild-type/Single mutations | 42 | 5 | 0.41 |
| *CBLC* |  |  |  |
| Mutant | 5 | 0 |  |
| Wild-type | 42 | 5 | 0.44 |
| *NF1* |  |  |  |
| Mutant | 3 | 1 |  |
| Wild-type | 44 | 4 | 0.28 |
| *CREBBP* |  |  |  |
| Mutant | 4 | 0 |  |
| Wild-type | 43 | 5 | 0.50 |
| *CBLB* |  |  |  |
| Mutant | 4 | 0 |  |
| Wild-type | 43 | 5 | 0.50 |
| *ATRX* |  |  |  |
| Mutant | 4 | 0 |  |
| Wild-type | 43 | 5 | 0.50 |
| *PPM1D* |  |  |  |
| Mutant | 2 | 2 |  |
| Wild-type | 45 | 3 | 0.004 |
| *PHF6* |  |  |  |
| Mutant | 3 | 0 |  |
| Wild-type | 44 | 5 | 0.56 |
| *TP53* |  |  |  |
| Mutant | 2 | 1 |  |
| Wild-type | 45 | 4 | 0.15 |
| *SMC1A* |  |  |  |
| Mutant | 3 | 0 |  |
| Wild-type | 44 | 5 | 0.56 |
| *NOTCH1* |  |  |  |
| Mutant | 3 | 0 |  |
| Wild-type | 44 | 5 | 0.56 |
| *KDM6A* |  |  |  |
| Mutant | 2 | 1 |  |
| Wild-type | 45 | 4 | 0.15 |
| *JAK3* |  |  |  |
| Mutant | 3 | 0 |  |
| Wild-type | 44 | 5 | 0.56 |
| *IDH1* |  |  |  |
| Mutant | 3 | 0 |  |
| Wild-type | 44 | 5 | 0.56 |
| *GNAS* |  |  |  |
| Mutant | 3 | 0 |  |
| Wild-type | 44 | 5 | 0.56 |
| *GATA2* |  |  |  |
| Mutant | 3 | 0 |  |
| Wild-type | 44 | 5 | 0.56 |
|  |  |  |  |

CR: complete remission; CRi: complete remission with incomplete hematological recovery; NR: non-remission; R1: first relapse; CR1: first complete remission

#: P-value is calculated by Pearson Chi-square test.
