# Supplementary material for: Clofarabine, cytarabine, and mitoxantrone in refractory/relapsed acute myeloid leukemia: High response rates and effective bridge to allogeneic hematopoietic stem cell transplantation
Source: Cancer Med. 2020 Mar 18;9(10):3371–82. doi: 10.1002/cam4.2865 (PMC7221314; doi:10.1002/cam4.2865)
Supplement: Supplementary file 8 [file CAM4-9-3371-s008.docx]

**Supplemental file 8. Outcome and toxicities following allogeneic hematopoietic stem cell transplantation in patients re-induced with CLAM.**

| **Parameter** | **Value** |
| --- | --- |
|  |  |
| Total number of patients^A^ | 24 |
|  |  |
| **HSCT charateristics** |  |
| **Status pre-HSCT** |  |
| CR/CRi following CLAM | 22 (92%) |
| NR following CLAM, CRi following decitabine | 1 (4%) |
| Relapse following CLAM, CRi following decitabine | 1 (4%) |
| **Donor** |  |
| HLA-matched sibling donors | 13 (54%) |
| Voluntary-unrelated donors | 11 (46%) |
| **Conditioning regimen** |  |
| Myeloablative conditioning (Bu-Cy) | 17 (71%) |
| Reduced-intensity conditioning (Flu-Bu) | 7 (29%) |
| **Median time to engraftment, days (range)** | 15 (10-21) |
|  |  |
| **Complications < 100 days post-HSCT** |  |
| Toxicity during conditioning | 2 (8%) |
| Seizures during Busulphan | 1 |
| Cardiotoxicity during Cyclophosphamide | 1 |
| **Acute GvHD** | 11 (46%) |
| Grade 1 acute GvHD skin | 2 |
| Grade 2 acute GvHD skin | 7 |
| Grade 3 acute GvHD skin, gut and liver | 2 |
| **Haemorrhagic cystititis** | 5 (21%) |
| **Thrombotic microangiopathy** | 1 (4%) |
| **Cyclosporin A-related acute kidney injury** | 1 (4%) |
| **Infections** | 6 (25%) |
| *S. viridans* pneumonia | 1 |
| *K. pneumoniae* bacteremia | 1 |
| *E. coli* bacteremia | 2 |
| *Serratia* bacteremia | 1 |
| Cytomegalovirus reactivation | 1 |
|  |  |
| **Complications ≥ 100 days post-HSCT** |  |
| **Chronic GvHD** | 7 (29%) |
| Mild chronic GvHD skin and sicca syndrome | 3 |
| Mild chronic GvHD liver and sicca syndrome | 2 |
| Moderate chronic GvHD skin and sicca syndrome | 1 |
| Severe chronic GvHD skin, sicca syndrome and lungs | 1 |
| **Infections** | 4 (17%) |
| Localized herpes zoster reactivation | 3 |
| Cytomegalovirus reactivation | 1 |
| **Relapse** | 5 (21%) |
| In patients failing CLAM prior to HSCT | 2/2 |
| CR/CRi after CLAM prior to HSCT | 3/22 |
| **Deaths** | 3 (13%) |
| Refractory AML | 2 |
| Deaths in remission following severe chronic GvHD lungs | 1 |
|  |  |

HSCT: hematopoietic stem cell transplantation; CR: complete remission; CRi: complete remission with incomplete hematological recovery; NR: non-remission; Bu-Cy: Busulphan-Cyclophosphamide; Flu-Bu: Fludarabine-Busulphan; GvHD: graft-*versus*-host disease; AML: acute myeloid leukemia; A: including 22 patients who underwent HSCT after initially achieving CR/CRi, 1 patient who had a relapse after CLAM-induced CRi, responded to salvage treatment and then underwent HSCT, and 1 patient who had NR to CLAM, responded to salvage treatment, and then underwent HSCT
