# Supplementary material for: Clofarabine, cytarabine, and mitoxantrone in refractory/relapsed acute myeloid leukemia: High response rates and effective bridge to allogeneic hematopoietic stem cell transplantation
Source: Cancer Med. 2020 Mar 18;9(10):3371–82. doi: 10.1002/cam4.2865 (PMC7221314; doi:10.1002/cam4.2865)
Supplement: Supplementary file 9 [file CAM4-9-3371-s009.docx]

**Supplemental file 9. Characteristics and outcome of patients with relapsed or refractory acute myeloid leukaemia failing CLAM.**

| **UPN/**  **Age/gender** | **Karyotype** | **Mutations at diagnosis** | **Status prior to CLAM (NR/R1)** | **Time to R1** | **NR to CLAM / Relapse (time to relapse)** | **Allogeneic HSCT before relapse** | **Treatment after NR/relapse** | **Outcome** |
| --- | --- | --- | --- | --- | --- | --- | --- | --- |
| UPN2  36/M | Normal | *ASXL1, CUX1, DNMT3A, IDH2, KIT, WT1* | NR | - | Relapse (27m) | Yes | HAG + PBSC rescue from original donor. Second HLA-matched sibling HSCT. | In CR2 |
| UPN3  38/M | del(11)(q23) | *ABL1, PTPN11, ROBO1, SETDB1, ZRSR2* | R1 | 2 | Relapse (2m) | No | Decitibine, HHT, Gemtuzumab ozogamacin | Death from refractory AML |
| UPN5  46/M | t(9;11)(p21;q23) | *CBL, CUX1, KMT2A, NPM1, PTPN11, ROBO1, SETD2, SMC3, TET2* | NR | - | Relapse (4m) | No | IT MTX, palliative care | Death from refractory AML |
| UPN6  37/M | t(8;21) -Y | *ASXL1, CBL, KIT, KMT2D, MPL, PTPN11, ROBO1, SETD2, SMC3, SRSF2, TERT* | R1 | 8 | Relapse (8m) | Yes | RT to myeloid sarcoma, Azacitidine + DLI | Death from severe cGVHD and bronchiolitis obliterans |
| UPN9  53/F | inv(3)(q21;q26), +X | *BCOR, CUX1, FLT3-ITD, KMT2A, ROBO1, SF3B1* | NR | - | Relapse (3m) | No | Azacitidine x 4 and achieved CR2, R1 26/1/17 given decitabine for 9 cycles and achieved CR3. HLA-matched sibling HSCT at CR3. Relapsed after HSCT and given venetoclax + decitabine | Death from refractory AML |
| UPN11  44/F | t(3;3)(q21;q26) | *ASXL1, CUX1, KMT2A, RUNX1* | NR | - | NR | - | Decitabine x 4 cycles with marrow blast count reduction (8%), followed by HLA-matched sibling HSCT | Relapsed 2 months post-allo HSCT and died from refractory AML |
| UPN12  36/M | t(8;21)(q22;q22),t(8;9),-Y | *ASXL1, CDKN2A, CUX1, KMT2D, SETDB1, SMC3, U2AF1* | R1 | 4 months | NR | - | ICE, Decitabine, Gemtuzumab ozogamacin | Death from refractory AML |
| UPN13  65/M | Normal | *FLT3-*ITD*, IDH2, NPM1, TET2* | R1 | 4 | Relapse (23m) | No | Decitabine | Death from refractory AML |
| UPN14  47/F | Normal | *ASXL1, CBLC, ETV6, FLT3-*ITD*, KMT2A, KMT2D, PTPN11, SETDB1, SRSF2, TET2, WT1* | NR | - | Relapse (4m) | No | Sorafenib + HHT | Death from refractory AML |
| UPN16  42/M | 93,XXYY[2]/XY[17] | *BCOR, CUX1, DNMT3A, FBXW7, IDH2, KMT2D, PHF6* | NR | - | NR | - | ICE, Venetoclax + decitabine | Death from refractory AML |
| UPN18  32/F | inv(3)(q21;q26) | *ASXL1, ATRX, BCOR, CBL, CUX1, EZH2, IDH1, HMT2B, PTPN11, RAD21, ROBO1, SETD2, SETDB1, SF3B1* | NR | - | Relapse (16m) | No | CLAM re-induction. Pending MUD HSCT | In CR2 |
| UPN17  35/F | Normal | *ACD, ASXL1, CDKN2A, CREBBP, CUX1, JAK3, KMT2A, MPL, RUNX1* | NR | - | Relapse (21m) | Yes | *FLT3*-ITD+ at relapse. Given Quizartinib + HHT | Refractory AML on palliative care |
| UPN20  65/M | inv(3)(q21;q26), monosomy 7 | *ASXL1, BCOR, DNMT3A, ETV6, RUNX1, ZRSR2* | NR | - | Relapse (3m) | No | Venetoclax + azacitidine | Death from refractory AML |
| UPN21  23/F | Normal | *EZH2, FLT3-*ITD*, RUNX1* | R1 | 16 | Relapse (5m) | No | *FLT3*-ITD+ at relapse. Given sorafenib + azacitidine | Death from refractory AML |
| UPN22  42/F | add(1)(p36.3),+8,der(8;17)(q10;q10),del(16)(p13.1) | *CBL, CUX1, HMT2D, PHF6, ROBO1, STAG2, TP53* | R1 | 2 months | NR | - | Decitabine | Death from refractory AML |
| UPN26  53/M | Normal | *ASXL1, CUX1, FLT3-*TKD*, KMT2A, RUNX1* | R1 | 15 | Relapse (4m) | No | Azacitidine | Death from refractory AML |
| UPN27  51/F | Normal | *BCOR, DNMT3A, NPM1, PPM1D, ROBO1, ROBO2, SMC3* | R1 | 11 | Relapse (2m) | No | HAG | Death from refractory AML |
| UPN28  63/F | Normal | *ASXL1, CREBBP, DNMT3A, EZH2, FLT3-ITD, IDH1, IDH2, NPM1, RUNX1, SETD2, TET2, U2AF1* | R1 | 28 | Relapse (6m) | No | Decitabine | Death from refractory AML |
| UPN33  62/F | del(9)(q13q22) | *CUX1, KMT2D, NRAS, PTPN11, RUNX1* | NR | - | Relapse (7m) | No | Decitabine | Refractory AML on palliative care |
| UPN39  35/F | t(8;21), del(7)(q32) | *FLT3-ITD, JAK3, KIT, KMT2B, KMT2D, NOTCH1, ROBO1, STAG2* | R1 | 12 | Relapse (7m) | No | ICE + Gemtuzumab ozogamacin | Refractory AML on palliative care |
| UPN41  49/M | t(8;21)(q22;q22) | *CBLB, CDKN2A, CREBBP, FLT3-*ITD*, IDH2, KMT2B, KMT2D, PDGFRA, SETDB1* | R1 | 8 | Relapse (2m) | No | ICE + Gemtuzumab ozogamacin, FLAG | Death from refractory AML |
| UPN43  37/F | inv(11)(q12q24) | *ASXL1, CBL, NF1, RAD21, ROBO2* | NR | - | NR | - | Decitabine | In CRi after 4 cycles of Decitabine, pending HLA-matched sibling HSCT |
| UPN45  30/F | inv(3)(q21;q26) | *ASXL1, CBLB, CUX1, DNMT3A, GNB1, NOTCH1, PTEN, RAD21, ROBO1, SF3B1, STAG2, TP53* | NR | - | Relapse (1m) | No | Venetoclax + decitabine, ICE + mylotarg, ATA | Death from refractory AML |
| UPN47  61/M | Normal | *ASXL1, BCORL1, DNMT3A, FLT3-TKD, IDH2, PHF6* | R1 | 10 | Relapse (2m) | No | Given venetoclax + Midostaurin + Decitabine | Refractory AML on palliative care |
| UPN48  22/M | Trisomy 19 | *ASXL1, CUX1, FLT3-*ITD*, NRAS, SETD2* | NR | - | Relapse (3m) | No | IT MTX, Craniospinal RT, HDAC | Death from refractory AML |
| UPN49  40/F | t(11;17)(p10;q10)[5] | *ASXL1, BCOR, ETV6, EZH2, FLT3-*ITD*, GATA2, KIT, NPM1, RAD21, RUNX1, SETDB1, TET2, WT1, ZRSR2* | R1 | 12 | Relapse (2m) | No | Palliative | Death from refractory AML |

CLAM: clofarabine, cytarabine (Ara-C), mitoxantrone; NR: non-remission; R1: first relapse; m: months; HSCT: haematopoietic stem cell transplantation; M: male; F: female; FLT3: fms-like tyrosine kinase-3; ITD: internal tandem duplication; TKD: tyrosine kinase domain; NPM1: nucleophosmin 1; HLA: human leucocyte antigen; ICE: idarubicin, cytarabine, etoposide; HAG: homoharrhingtonin, cytarabine (Ara-C), G-CSF; FLAG: fludarabine, cytarabine (Ara-C), G-CSF; ATA: amsacrine; thioguanine; cytarabine (Ara-C); IT: intrathecal; MTX: methotrexate; RT: radiotherapy; DLI: donor lymphocyte infusion; HDAC: high-dose cytarabine (Ara-C); AML: acute myeloid leukaemia; CRi: complete remission with incomplete haematological recovery; CR2: second complete remission; CR3: third complete remission.
