# Supplementary material for: Clofarabine, cytarabine, and mitoxantrone in refractory/relapsed acute myeloid leukemia: High response rates and effective bridge to allogeneic hematopoietic stem cell transplantation
Source: Cancer Med. 2020 Mar 18;9(10):3371–82. doi: 10.1002/cam4.2865 (PMC7221314; doi:10.1002/cam4.2865)
Supplement: Supplementary file 10 [file CAM4-9-3371-s010.docx]

**Supplemental file 10. Prognostic impact of gene mutations on survival in patients re-induced with CLAM.**

|  |  | **Overall survival** | | |  |  |  | **Relapse-free survival** | | |  |  |  | **Event-free survival** | | |  |  |
| --- | --- | --- | --- | --- | --- | --- | --- | --- | --- | --- | --- | --- | --- | --- | --- | --- | --- | --- |
| **Gene^A^** | **No.** | **2-year** | **HR (95% CI)** | **P-value** | | |  | **2-year** | **HR (95% C.I.)** | **P-value** | | |  | **2-year** | **HR (95% CI)** | **P-value** | | |
| *CUX1* |  |  |  |  | | |  |  |  |  | | |  |  |  |  | | |
| mutant | 31 | 73.7% | 0.45 (0.17-1.18) | 0.10 | | |  | 53.2% | 0.38 (0.16-0.91) | 0.03 | | |  | 53.0% | 0.46 (0.21-1.07) | 0.05 | | |
| wildtype | 21 | 40.0% | 2.23 (0.85-5.83) |  | | |  | 20.1% | 2.65 (1.10-6.40) |  | | |  | 19.1% | 2.18 (0.99-4.77) |  | | |
| *ASXL1* |  |  |  |  | | |  |  |  |  | | |  |  |  |  | | |
| mutant | 28 | 60.9% | 1.02 (0.39-2.67) | 0.97 | | |  | 20.9% | 1.38 (0.57-3.35) | 0.48 | | |  | 18.4% | 1.36 (0.61-3.01) | 0.45 | | |
| wildtype | 24 | 63.4% | 0.98 (0.38-2.56) |  | | |  | 51.3% | 0.73 (0.30-1.77) |  | | |  | 44.3% | 0.74 (0.33-1.64) |  | | |
| *KMT2D* |  |  |  |  | | |  |  |  |  | | |  |  |  |  | | |
| mutant | 19 | 67.0% | 0.84 (0.31-2.28) | 0.73 | | |  | 65.6% | 0.48 (0.18-1.32) | 0.16 | | |  | 52.1% | 0.63 (0.27-1.47) | 0.28 | | |
| wildtype | 33 | 59.6% | 1.19 (0.44-3.24) |  | | |  | 23.0% | 2.08 (0.77-5.71) |  | | |  | 50.9% | 1.59 (0.69-3.67) |  | | |
| *ROBO1* |  |  |  |  | | |  |  |  |  | | |  |  |  |  | | |
| mutant | 16 | 68.8% | 1.74 (0.66-4.60) | 0.26 | | |  | 35.6% | 1.64 (0.67-4.01) | 0.28 | | |  | 33.3% | 1.56 (0.69-3.53) | 0.29 | | |
| wildtype | 36 | 60.2% | 0.57 (0.22-1.51) |  | | |  | 43.4% | 0.61 (0.25-1.48) |  | | |  | 36.7% | 0.64 (0.28-1.45) |  | | |
| *RUNX1* |  |  |  |  | | |  |  |  |  | | |  |  |  |  | | |
| mutant | 15 | 46.7% | 1.27 (0.47-3.43) | 0.64 | | |  | 38.1% | 1.15 (0.46-2.84) | 0.77 | | |  | 34.3% | 1.04 (0.45-2.38) | 0.93 | | |
| wildtype | 37 | 70.4% | 0.79 (0.29-2.14) |  | | |  | 39.9% | 0.87 (0.35-2.17) |  | | |  | 33.8% | 0.97 (0.42-2.22) |  | | |
| *FLT3* |  |  |  |  | | |  |  |  |  | | |  |  |  |  | | |
| ITD | 14 | 53.6% | 1.64 (0.60-4.48) | 0.33 | | |  | 0% | 2.19 (0.91-5.24) | 0.08 | | |  | 0% | 1.74 (0.77-3.92) | 0.18 | | |
| Non-ITD | 38 | 66.3% | 0.61 (0.22-1.66) |  | | |  | 50.1% | 0.46 (0.19-1.20) |  | | |  | 41.2% | 0.58 (0.26-1.30) |  | | |
| *SETBB1* |  |  |  |  | | |  |  |  |  | | |  |  |  |  | | |
| mutant | 14 | 58.9% | 1.28 (0.45-3.64) | 0.64 | | |  | 55.4% | 0.85 (0.31-2.33) | 0.76 | | |  | 51.4% | 0.86 (0.35-2.16) | 0.76 | | |
| wildtype | 38 | 62.9% | 0.78 (0.27-2.22) |  | | |  | 36.0% | 1.17 (0.43-3.20) |  | | |  | 30.4% | 1.16 (0.46-2.88) |  | | |
| *DNMT3A* |  |  |  |  | | |  |  |  |  | | |  |  |  |  | | |
| mutant | 13 | 35.3% | 1.39 (0.49-3.99) | 0.54 | | |  | 0% | 1.55 (0.59-4.04) | 0.37 | | |  | 0% | 1.38 (0.58-3.32) | 0.47 | | |
| wildtype | 39 | 70.1% | 0.72 (0.25-2.05) |  | | |  | 45.4% | 0.65 (0.25-1.69) |  | | |  | 40.6% | 0.72 (0.30-1.74) |  | | |
| *KMT2A* |  |  |  |  | | |  |  |  |  | | |  |  |  |  | | |
| mutant | 11 | 59.7% | 1.63 (0.57-4.66) | 0.36 | | |  | 30.0% | 1.31 (0.47-3.63) | 0.61 | | |  | 26.5% | 1.22 (0.48-3.07) | 0.68 | | |
| wildtype | 41 | 63.4% | 0.61 (0.21-1.75) |  | | |  | 44.1% | 0.77 (0.28-2.13) |  | | |  | 38.0% | 0.82 (0.33-2.07) |  | | |
| *PTPN11* |  |  |  |  | | |  |  |  |  | | |  |  |  |  | | |
| mutant | 11 | 68.2% | 1.19 (0.17-2.09) | 0.76 | | |  | 26.5% | 1.42 (0.55-3.69) | 0.47 | | |  | 26.5% | 1.25 (0.50-3.13) | 0.64 | | |
| wildtype | 41 | 61.3% | 0.84 (0.27-2.58) |  | | |  | 42.6% | 0.70 (0.27-1.83) |  | | |  | 60.1% | 0.80 (0.32-2.01) |  | | |
| *SETD2* |  |  |  |  | | |  |  |  |  | | |  |  |  |  | | |
| mutant | 11 | 51.9% | 0.59 (0.17-2.09) | 0.42 | | |  | 50.9% | 0.86 (0.32-2.35) | 0.77 | | |  | 50.9% | 0.74 (0.28-1.97) | 0.55 | | |
| wildtype | 41 | 56.5% | 1.68 (0.48-5.92) |  | | |  | 36.1% | 1.16 (0.43-3.18) |  | | |  | 29.4% | 1.35 (0.51-3.59) |  | | |
| *IDH2* |  |  |  |  | | |  |  |  |  | | |  |  |  |  | | |
| mutant | 10 | 45.0% | 0.91 (0.26-3.19) | 0.89 | | |  | 0% | 1.36 (0.50-3.73) | 1.36 | | |  | 0% | 1.23 (0.49-3.06) | 0.66 | | |
| wildtype | 42 | 64.7% | 1.09 (0.31-3.82) |  | | |  | 51.2% | 0.73 (0.27-2.01) |  | | |  | 45.8% | 0.82 (0.33-2.04) |  | | |
| *TET2* |  |  |  |  | | |  |  |  |  | | |  |  |  |  | | |
| mutant | 10 | 57.1% | 1.38 (0.44-4.37) | 0.52 | | |  | 0% | 1.17 (0.42-3.16) | 0.78 | | |  | 0% | 0.96 (0.36-2.56) | 0.94 | | |
| wildtype | 42 | 63.7% | 0.72 (0.23-2.28) |  | | |  | 44.5% | 0.87 (0.32-2.37) |  | | |  | 36.6% | 1.04 (0.39-2.76) |  | | |
| *BCOR* |  |  |  |  | | |  |  |  |  | | |  |  |  |  | | |
| mutant | 10 | 53.3% | 1.59 (0.55-4.56) | 0.39 | | |  | 37.0% | 1.87 (0.66-5.24) | 0.24 | | |  | 30.0% | 1.61 (0.63-4.09) | 0.32 | | |
| wildtype | 42 | 66.7% | 0.63 (0.22-1.81) |  | | |  | 44.2% | 0.54 (0.19-1.51) |  | | |  | 58.2% | 0.62 (0.24-1.58) |  | | |
|  |  |  |  |  | | |  |  |  |  | | |  |  |  |  | | |

OS: overall survival; RFS: relapse-free survival; EFS: event-free survival; HR: hazard ratio for event; CI: confidence interval.

A: Only genes mutated in 10 or more patients are shown.
