# Supplementary material for: Clofarabine, cytarabine, and mitoxantrone in refractory/relapsed acute myeloid leukemia: High response rates and effective bridge to allogeneic hematopoietic stem cell transplantation
Source: Cancer Med. 2020 Mar 18;9(10):3371–82. doi: 10.1002/cam4.2865 (PMC7221314; doi:10.1002/cam4.2865)
Supplement: Supplementary file 11 [file CAM4-9-3371-s011.docx]

**Supplemental file 11. Clinicopathologic features, karyotypic changes and gene mutations in patients receiving and not receiving allogeneic hematopoietic stem cell transplantation**

|  | **Number of patients** | |  |
| --- | --- | --- | --- |
|  | **HSCT** | **Non-HSCT** | **P value** |
| Total | 22 | 30 |  |
| **Gender** |  |  |  |
| Male | 12 | 14 |  |
| Female | 10 | 16 | 0.58 |
| **Age** |  |  |  |
| 18-45 years | 9 | 15 |  |
| 46-65 years | 13 | 15 | 0.52 |
| **Status** |  |  |  |
| relapse | 15 | 16 |  |
| refractory | 7 | 14 | 0.28 |
| **Response** |  |  |  |
| CR | 20 | 16 |  |
| CRi | 2 | 9 |  |
| NR | 0 | 5 | 0.01 |
| **Karyotype** |  |  |  |
| Normal | 15 | 10 |  |
| Core-binding factor AML: t(8;21)(q22;q22.1);  inv(16)(p13.1q22)/t(16;16)(p13.1;q22) | 3 | 6 |  |
| t(9;11)(p21.2;q23.3) | 0 | 1 |  |
| inv(3)(q21.3q26.2)/t(3;3)(q21.2;q23.3) | 1 | 5 |  |
| t(v;11q23.3) / del(11)(q23) | 1 | 1 |  |
| Complex | 0 | 2 |  |
| Others | 2 | 5 | 0.24 |
| **Gene mutations** |  |  |  |
| *CUX1* |  |  |  |
| mutant | 17 | 14 |  |
| wildtype | 5 | 16 | 0.03 |
| *ASXL1* |  |  |  |
| mutant | 13 | 15 |  |
| wildtype | 9 | 15 | 0.52 |
| *KMT2D* |  |  |  |
| mutant | 7 | 12 |  |
| wildtype | 15 | 18 | 0.55 |
| *ROBO1* |  |  |  |
| mutant | 7 | 9 |  |
| wildtype | 15 | 21 | 0.89 |
| *RUNX1* |  |  |  |
| mutant | 8 | 7 |  |
| wildtype | 14 | 23 | 0.31 |
| *FLT3* |  |  |  |
| ITD | 5 | 9 |  |
| Non-ITD | 17 | 21 | 0.56 |
| *SETDB1* |  |  |  |
| mutant | 7 | 7 |  |
| wildtype | 15 | 23 | 0.50 |
| *DNMT3A* |  |  |  |
| mutant | 7 | 6 |  |
| wildtype | 15 | 24 | 0.33 |
| *KMT2A* |  |  |  |
| mutant | 5 | 6 |  |
| wildtype | 17 | 24 | 0.81 |
| *PTPN11* |  |  |  |
| mutant | 5 | 6 |  |
| wildtype | 17 | 24 | 0.81 |
| *SETD2* |  |  |  |
| mutant | 7 | 4 |  |
| wildtype | 15 | 26 | 0.11 |
| *IDH2* |  |  |  |
| mutant | 5 | 5 |  |
| wildtype | 17 | 25 | 0.58 |
| *TET2* |  |  |  |
| mutant | 3 | 7 |  |
| wildtype | 19 | 23 | 0.38 |
| *BCOR* |  |  |  |
| mutant | 3 | 7 |  |
| wildtype | 19 | 23 | 0.38 |
| *CEBPA* |  |  |  |
| double mutations | 4 | 1 |  |
| wildtype/single mutations | 18 | 29 | 0.07 |
| *IDH1* |  |  |  |
| mutant | 1 | 2 |  |
| wildtype | 21 | 28 | 0.75 |
| *NPM1* |  |  |  |
| mutant | 2 | 7 |  |
| wildtype | 20 | 23 | 0.18 |
| *TP53* |  |  |  |
| mutant | 1 | 2 |  |
| wildtype | 21 | 28 | 0.74 |
| *U2AF1* |  |  |  |
| mutant | 0 | 2 |  |
| wildtype | 22 | 28 | 0.22 |
|  |  |  |  |
