# Supplementary material for: Clofarabine, cytarabine, and mitoxantrone in refractory/relapsed acute myeloid leukemia: High response rates and effective bridge to allogeneic hematopoietic stem cell transplantation
Source: Cancer Med. 2020 Mar 18;9(10):3371–82. doi: 10.1002/cam4.2865 (PMC7221314; doi:10.1002/cam4.2865)
Supplement: Supplementary file 12 [file CAM4-9-3371-s012.docx]

**File S1.** CLAM protocol at Queen Mary Hospital, Hong Kong.

**File S2.** Methodology for next-generation sequencing.

**File S3.** Karyotypes of 52 patients with relapsed or refractory acute myeloid leukemia.

**File S4.** Frequency of gene mutations at diagnosis in 52 patients with relapsed or refractory acute myeloid leukemia. A. Horizontal bar chart shows the frequency of mutations in 58 mutated genes. The vertical axis represents the genes mutated and the horizontal axis shows the number of patients with mutations in that gene. B. Pie chart shows the proportion of patients with different numbers of gene mutations (1, 2-4, 5-7, 8-10, 11 or more). C. Horizontal bar chart shows the frequency of mutations in each functional category. The vertical axis represents the functional category of genes and the horizontal axis shows the number of patients with mutations in that functional category.

**File S5.** Frequency of mutations in each functional category at diagnosis in 52 patients with relapsed or refractory acute myeloid leukemia treated with CLAM. The vertical axis represents the functional category of genes and the horizontal axis shows the number of patients with mutations in that functional category. The number of patients with mutations in specific genes in each functional category is shown in each stacked horizontal bar.

**File S6.** Circos plot shows concurrent gene mutations at diagnosis in 52 patients with relapsed/refractory acute myeloid leukemia treated with CLAM. Each connection represents the concurrence of mutations in two genes in a patient. Only mutations present in more than 2 patients are shown.

**File S7.** Prognostic factors for response in 52 patients with relapsed or refractory AML treated with CLAM.

**File S8.** Outcome and toxicities following allogeneic hematopoietic stem cell transplantation in patients reinduced with CLAM.

**File S9.** Characteristics and outcome of patients with relapsed or refractory acute myeloid leukemia failing CLAM.

**File S10.** Prognostic impact of gene mutations on survival in patients reinduced with CLAM.

**File S11.** Clinicopathologic features, karyotypic changes, and gene mutations in patients receiving and not receiving allogeneic hematopoietic stem cell transplantation
